# Cyclin D1 integrates G9a-mediated histone methylation

**DOI:** 10.1038/s41388-019-0723-8

**Published:** 2019-02-04

**Authors:** Zhiping Li, Xuanmao Jiao, Gabriele Di Sante, Adam Ertel, Mathew C. Casimiro, Min Wang, Sanjay Katiyar, Xiaoming Ju, D. V. Klopfenstein, Aydin Tozeren, William Dampier, Iouri Chepelev, Albert Jeltsch, Richard G. Pestell

**Affiliations:** 1 Pennsylvania Cancer and Regenerative Medicine Research Center, Baruch S. Blumberg Institute, Pennsylvania Biotechnology Center, 3805 Old Easton Rd., Doylestown, PA 18902 USA; 2 Department of Cancer Biology, Thomas Jefferson University, 233 South 10th Street, Philadelphia, PA 19107 USA; 3 Center for Integrated Bioinformatics, School of Biomedical Engineering, Drexel University, Philadelphia, PA 19104 USA; 4 Department of Microbiology & Immunology, Drexel University College of Medicine, Philadelphia, PA 19104 USA; 5 Center for Autoimmune Genomics and Etiology, Cincinnati Children’s Hospital Medical Center, Cincinnati, OH 45229 USA; 6 Department of Pediatrics, University of Cincinnati College of Medicine, Cincinnati, OH 45267 USA; 7 Department of Biochemistry, Institute of Biochemistry and Technical Biochemistry, University of Stuttgart, Allmandring 31, D-70569 Stuttgart, Germany; 8 Lee Kong Chian School of Medicine, Nanyang Technological University, Singapore, 637551 Singapore

**Keywords:** Checkpoint signalling, Breast cancer

## Abstract

Lysine methylation of histones and non-histone substrates by the SET domain containing protein lysine methyltransferase (KMT) G9a/EHMT2 governs transcription contributing to apoptosis, aberrant cell growth, and pluripotency. The positioning of chromosomes within the nuclear three-dimensional space involves interactions between nuclear lamina (NL) and the lamina-associated domains (LAD). Contact of individual LADs with the NL are dependent upon H3K9me2 introduced by G9a. The mechanisms governing the recruitment of G9a to distinct subcellular sites, into chromatin or to LAD, is not known. The *cyclin D1* gene product encodes the regulatory subunit of the holoenzyme that phosphorylates pRB and NRF1 thereby governing cell-cycle progression and mitochondrial metabolism. Herein, we show that cyclin D1 enhanced H3K9 dimethylation though direct association with G9a. Endogenous cyclin D1 was required for the recruitment of G9a to target genes in chromatin, for G9a-induced H3K9me2 of histones, and for NL-LAD interaction. The finding that cyclin D1 is required for recruitment of G9a to target genes in chromatin and for H3K9 dimethylation, identifies a novel mechanism coordinating protein methylation.

## Introduction

Histone methylation is dynamically regulated by histone methyltransferases (HKMTs) and histone lysine demethylases [1]. Both histone and non-histone substrates have been reported for HKMTs therefore these enzymes are referred to as lysine methyltransferases (KMTs) and lysine demethylases. The key KMTs include G9a/KMT1C, which methylates histone H1 and H3 (K9 and K27) in vitro. The Su(var)3-9-Enhancer of *zeste*-Trithorax (SET) domain of Suv39h1/KMT1a encodes the catalytic domain, which governs lysine methylation [2]. Both Suv39h1 and G9a catalyze mono-, di-, and tri-methylation reactions on H3K9 [3, 4]. In mouse and human, the G9a/GLP enzymatic complex di-methylates H3K9. G9a is therein essential for both the stability of the complex and the catalytic function [5]. G9a associates with heterochromatin protein 1 (HP1) to regulate chromatin binding and association with methylated histones [6]. Distinct domains of G9a, including the Cys-rich region (CYS), the ankyrin repeat (ANK), and the SET domain, facilitate interactions with either methylated histones or associated proteins. The recruitment of HP1α and HP1β to pericentromeric heterochromatin is dependent upon H3K9 methylation by Suv39h. HP1α then binds to H3K9me2 and H3K9me3. The association of G9a with HP1 to form complexes increases the automethylation of G9a [6–8]. The association of the multi H3K9 methyl-binding protein modules, which includes HP1 and G9a, may result in the spreading of H3K9me2 marks [9]. The non-histone substrates of G9a include p53, Wiz, CDYL1, ACINUS, and Reptin [10–13].

The nuclear lamina (NL) interacts with genomic regions referred to as lamina-associated domains (LADs) and G9a plays a critical role in NL-associated large chromatin domain interactions. Recent evidence suggests the contact of NL with LADs is linked to H3K9 dimethylation introduced by G9a [14]. G9a thereby contributes to architectural changes of chromosomes and in turn participates in gene regulation. Approximately 40% of the mammalian genome are covered by LADs. LAD interactions with NL may provide structural impediments that positioning the chromosomes. The mechanisms governing LAD-NL association is contingent upon long (GA)_*n*_ repeats [15]. Furthermore, H3K9 methylation contributes to NL anchoring to genomic loci [16]. The mechanisms coordinating the interactions between LAD and NL remain to be determined. Because a variety of diseases have been linked to dysfunctional interaction of NL-associated proteins and chromatin components [17], it remains important to discern the mechanism governing these interactions.

Recently, a new approach was developed for studying NL-LAD interactions using an epigenetic tag of DNA adenine-6-methylation. DNA in contact with the NL becomes adenine methylated via an *Escherichia coli* DNA adenine methyltransferase and Lamin B fusion protein. Because ^m6^A is a stable modification, DNA in contact with the NL can be labeled and thereby visualized in living cells. Using this approach, G9a was shown to control H3K9 dimethylation, which was critical for LAD-NL interactions and thereby determined the contact of LADs with the NL [14]. G9a is known to colocalize with the replication foci during DNA synthesis, and shortly prior to incorporation into chromatin, G9a complexes deposit K9me2 marks on H3 [18, 19].

The *cyclin D1* gene encodes a labile regulatory subunit of the holoenzyme that phosphorylates and inactivates the retinoblastoma (pRb) [20] and NRF1 [21] proteins thereby coordinating both the DNA synthetic phase of the cell cycle and mitochondrial biogenesis [22]. Several recent studies have implicated cyclin D1 in the regulation of gene transcription [23]. Initial studies demonstrated cyclin D1 altered both transcription factor recruitment and local chromatin acetylation in chromatin immunoprecipitation (ChIP) assays [24]. Such findings were consistent with the binding of cyclin D1 to histone acetylases and deacetylases in vitro [25–28]. Cyclin D1 was subsequently identified in a DNA chromatin-associated pool linked to the regulation of gene expression, including the repression of PPARγ [27, 29] and unbiased genome-wide ChIP-Seq demonstrated cyclin D1 binds to the regulatory regions of genes governing chromosomal instability [30]. Cyclin D1 is known to either activate or repress gene expression, and more than 30 transcription factors and several co-activators interacting with cyclin D1 have been characterized. The regulation of gene expression by cyclin D1 involves a helix-turn-helix (HTH) domain between aa179 and 241 [27]. The biological significance of endogenous cyclin D1 in governing gene expression in vivo was evidenced by recent studies in which *cyclin D1* genetic deletion attenuated both estradiol- and androgen-dependent gene expression in the mammary gland [31] and prostate, respectively [32].

We show herein that cyclin D1 governs H3K9 dimethylation of histone substrates and determines the recruitment of G9a into chromatin at gene targets. Cyclin D1 enhanced H3K9me2 in tissue culture and in vivo in multigenic mice. Endogenous cyclin D1 bound the predominant cellular H3K9 methyltransferase G9a. The previously defined HTH transcriptional regulatory domain of cyclin D1 was required for association with G9a. Cyclin D1 binding to G9a required the CYS domain of G9a. Cyclin D1 and G9a bound common genes in genome-wide ChIP-Seq analyses, with enrichment at the LAD borders. Using ^m6^A-tracer, we show cyclin D1 is required for the G9a-dependent association of NL with LAD. Collectively, these studies define a novel function for cyclin D1, to associate with G9a and thereby promote H3K9 dimethylation, which in turn plays an essential role in the positioning of interphase chromosomes.

## Results

### The cyclin D1 HTH domain is required for binding to G9a

**Fig. 1:**
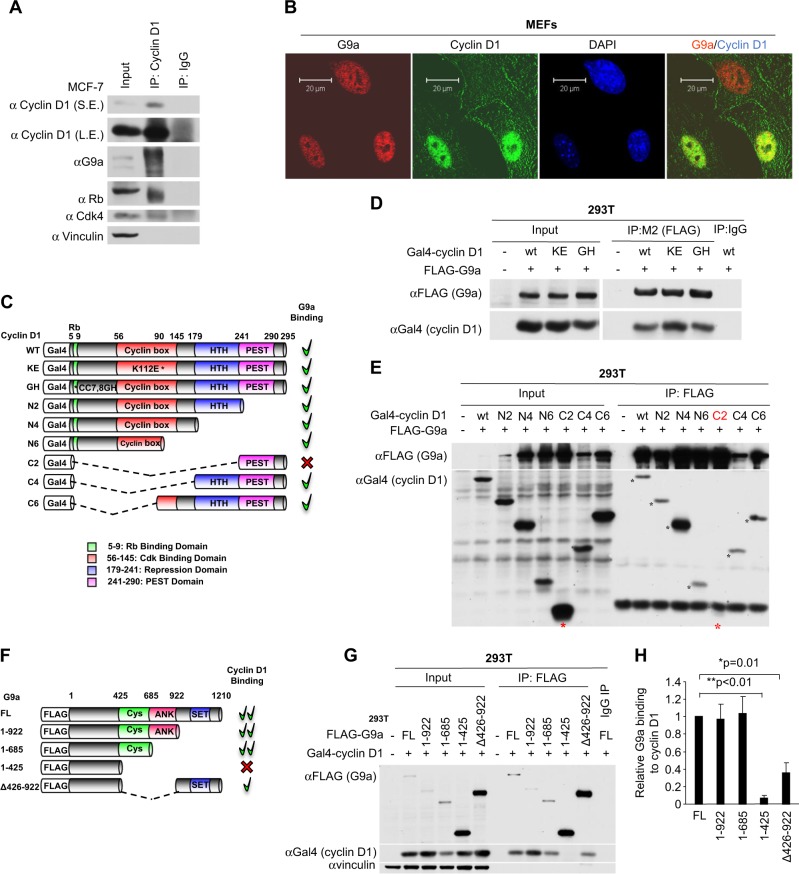
Cyclin D1 binds G9a. **a** Cyclin D1 immune-precipitation was conducted in MCF-7 cells, with subsequent western blotting to the proteins indicated. **b** Confocal microscopy of immunofluorescence for G9a (red), cyclin D1 (green), and nuclear staining with 4′,6-diamidino-2-phenylindole (DAPI; blue) in *cyclin D1*^*+/+*^ mouse embryonic fibroblasts (MEFs). Scale bar, 20 μm. **c** Schematic representation of GAL4-tagged cyclin D1 expression vectors with relative G9a binding. **d** Immunoprecipitation using an antibody to the FLAG-tag of G9a with sequential western blot to the GAL4 for GAL4-cyclin D1 in 293T cells co-transfected with FLAG-tagged G9a and GAL4-tagged cyclin D1 wild type, KE mutant, and GH mutant. **e** Immunoprecipitation using an antibody to the FLAG-tag of G9a and sequential western blotting to GAL4 for GAL4-cyclin D1 wild type and truncation mutants. Note the relative abundance of C2 and C4 in the input and lack of cyclin D1 in C2 but not in C4 by FLAG immunoprecipitation (IP)-western blot. **f** Schematic representation of FLAG-tagged G9a expression vectors with relative cyclin D1 binding. **g** IP-western after precipitation with anti-FLAG antibody for FLAG-tagged G9a in sequential western blotting to the GAL4 for GAL4-cyclin D1 in 293T cells co-transfected with GAL4-tagged cyclin D1 and FLAG-tagged G9a wild type and truncation mutants. **h** Quantitation of FLAG-tagged G9a binding to GAL4-tagged cyclin D1 in 293T cells from three independent experiments. The binding amount of FLAG-tagged wild-type G9a to GAL4-tagged cyclin D1 in each experiment was set as 1. The data are shown as mean ± SEM. *P = 0.01; **P < 0.01 (n = 3)

The HMT G9a is responsible for the majority of H3K9me2 in cells. In order to determine whether the induction of H3K9me2 at specific chromatin elements by cyclin D1 involved association of cyclin D1 with G9a, immune-precipitation was conducted of endogenous cyclin D1 in the human MCF-7 breast cancer cell line. Cyclin D1 immunoprecipitation (IP) co-precipitated pRB, Cdk4, and G9a (Fig. 1a). Immunofluorescence staining suggested that cyclin D1 (green) co-localized with G9a (red) in wild-type mouse embryonic fibroblasts (MEFs) in a subset of cells (Fig. 1b, yellow dots).

In order to determine which domain of cyclin D1 binds to G9a we conducted IP-western blotting of cells transfected with expression vectors encoding GAL4-tagged cyclin D1 or mutants of cyclin D1 (Fig. 1c) and a vector encoding FLAG-tagged wild-type G9a (Fig. 1d, e). IP with an anti-FLAG antibody precipitated FLAG-tagged G9a and the associated GAL4-cyclin D1 fusion protein. Point mutation of cyclin D1 at the amino acid K112, which is known to reduce binding of Cdk4/Cdk6, did not affect G9a association (Fig. 1d). As the cyclin D1^KE^ mutant is defective in forming an active kinase function, this finding suggests G9a binding is distinct from the kinase function of cyclin D1. Similarly, the cyclin D1^GH^ mutant, which abrogates pRb binding, did not affect G9a association (Fig. 1d). We next examined the series of deletion mutants of cyclin D1. The N6 mutant was capable of binding G9a, however, the C2 mutant, which was expressed abundantly, failed to bind G9a (Fig. 1e, asterisks). The C4 and C6 mutants of cyclin D1 bound G9a. Because the amino terminal fragment of cyclin D1 (N6) and the C-terminal fragment (C4) were capable of binding G9a together, these studies demonstrate that the amino terminus and the amino-acid residues between 178 and 242, corresponding to the previously described cyclin D1 transcriptional regulatory domain [27], are both required for G9a binding in IP-western blotting.

Quantitation of the relative association between G9a and cyclin D1 was conducted. In order to define the domains of G9a required for cyclin D1 association in IP-western blotting, a series of G9a expression vectors were deployed (Fig. 1f). The FLAG-tagged G9a expression vectors were co-expressed with cyclin D1^WT^. The FLAG IP precipitated G9a and the co-associated cyclin D1 was detected by western blot (Fig. 1g). The G9a mutant 1-425 failed to bind cyclin D1 (Fig. 1g), whereas the G9a mutant 1-685 including the CYS domain was capable of binding cyclin D1 (Fig. 1g). The G9a mutant Del 426-922 without the CYS and ANK domains significantly decreased binding to cyclin D1 (Fig. 1g, h, G9a FL vs 1-425, *P* < 0.01; G9a FL vs Del 426-922, *P* = 0.01) (*n* = 3). Together, these studies demonstrate that the G9a CYS domain and the cyclin D1 HTH domain are required for co-association.

### Cyclin D1 maintains H3K9 dimethylation in cultured cells and in vivo

**Fig. 2:**
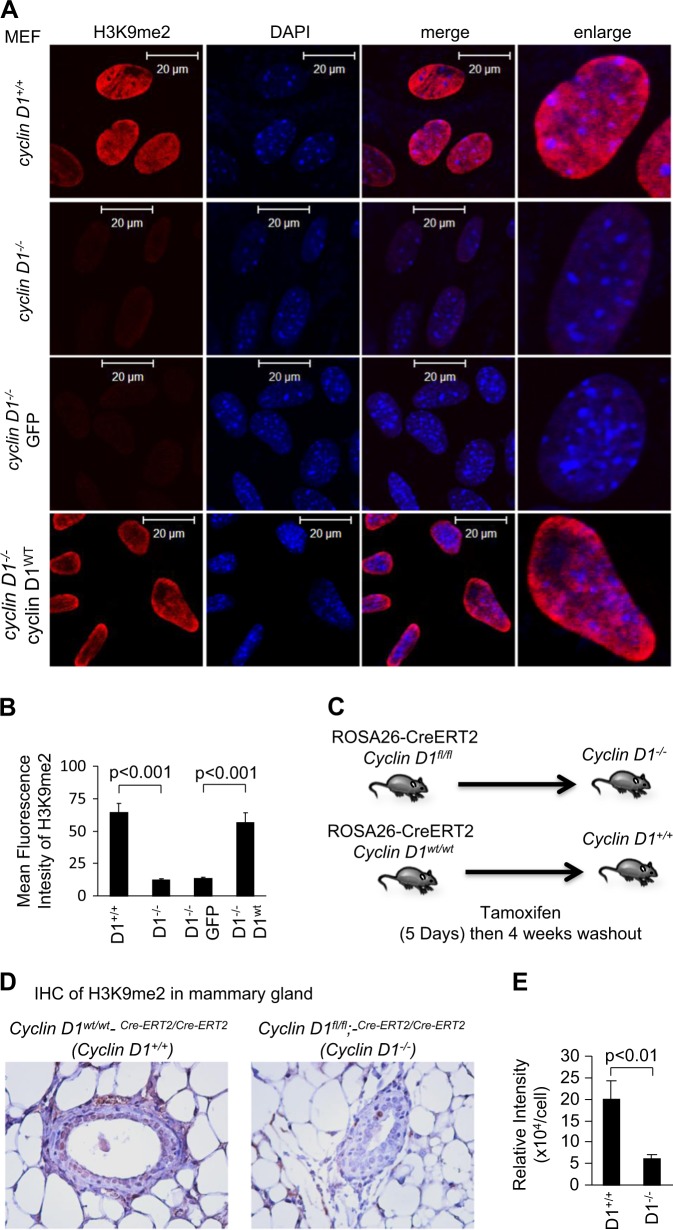
Cyclin D1 augments H3K9me2. **a** Confocal microscopy of immunofluorescence for H3K9me2 (red) and nuclear staining with 4′,6-diamidino-2-phenylindole (DAPI; blue) in cyclin D1 wild-type and knockout mouse embryonic fibroblasts (MEFs), and *cyclin D1*^*−/−*^ MEFs rescued with MSCV-cyclin D1-IRES-GFP or vector control. Images demonstrate the reduction in H3K9me2 in *cyclin D1*^*−/−*^ cells. Scale bar, 20 μm with (**b**) quantitation of mean fluorescence shown as mean ± SEM. **c** Schematic representation of transgenic paradigm. **d** Immunohistochemical staining for H3K9me2 in the mammary gland of transgenic mice in which the *cyclin D1* gene was deleted through Cre excision in the adult mammary glands. **e** The quantitation of H3K9me2 is shown as mean ± SEM for *n* = 10 separate mammary glands from two *cyclin D1*^*WT*^ (tamoxifen-treated *cyclin D1*^*wt/wt*^*;-Rosa26*^*CreERT2/CreERT2*^ transgenic mice) and three *cyclin D1*^*−/−*^ mice (tamoxifen-treated *cyclin D1*^*fl/fl*^*;-Rosa26*^*CreERT2/CreERT2*^ transgenic mice)

In order to determine the role of endogenous cyclin D1 in determining cellular H3K9me2, immunofluorescence was conducted. We examined H3K9me2, comparing the *cyclin D1*^*+*/*+*^, *cyclin D1*^*−*/−^ MEFs, and *cyclin D1*^*−*/−^ MEFs rescued with cyclin D1 or vector control (Fig. 2a, b). The relative abundance of H3K9me2 was reduced in *cyclin D1*^−/−^ cells compared to wild-type controls. Introducing cyclin D1 into *cyclin D1*^*−/−*^ MEFs increased H3K9me2 compared to the vector control cells (Fig. 2a, b). Introducing a point mutation of the amino acid K112 (KE mutant), which results in reduced binding of cdk4/cdk6 and impaired kinase activity when introduced into *cyclin D1*^−/−^ MEFs, also enhanced H3K9me2 staining to a similar level as cyclin D1 wild type (Supplemental Fig. 1).

In order to determine whether endogenous cyclin D1 maintained H3K9me2 in vivo, transgenic mice were used. *Cyclin D1*^*fl/fl*^ mice were intercrossed with *Rosa26*^*CreERT2/CreERT2*^ transgenic mice, and adult mice were treated with tamoxifen to induce Cre expression and thereby *cyclin D1* gene deletion (Fig. 2c). After 4 weeks of tamoxifen washout, immunohistochemical staining of H3K9me2 and cyclin D1 was conducted, demonstrating a substantial reduction in H3K9me2 upon deletion of the *cyclin D1* gene (Fig. 2d, e). *Cyclin D1* gene deletion was verified in these mice by genomic analysis and by immunohistochemical staining for cyclin D1 (Supplemental Fig. 2).

### The cyclin D1 HTH domain is required for induction of H3K9me2

**Fig. 3:**
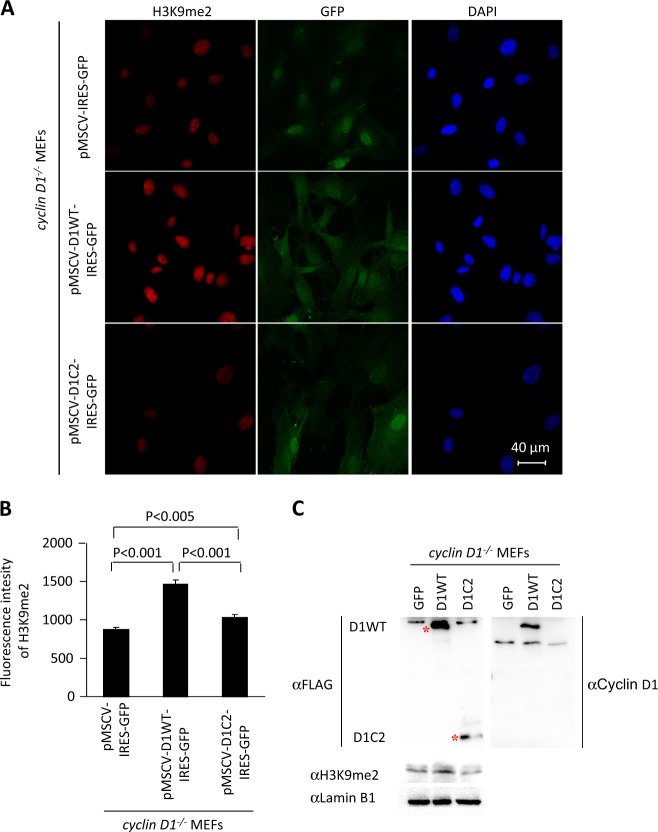
The G9a-binding defective mutant of cyclin D1 fails to augment H3K9me2. **a** Confocal microscopy of immunofluorescence for H3K9me2 (red) and nuclear staining with 4′,6-diamidino-2-phenylindole (DAPI; blue) in cyclin D1 wild-type and knockout mouse embryonic fibroblasts (MEFs), and *cyclin D1*^*−/−*^ MEFs rescued with MSCV-cyclin D1^WT^-IRES-GFP, MSCV-cyclin D1^C2^-IRES-GFP, or vector control. Images demonstrate the reduction in H3K9me2 in *cyclin D1*^*−/−*^ cells and rescue with cyclin D1^WT^. Scale bar, 40 μm with (**b**) quantitation of mean fluorescence shown as mean ± SEM. **c** Western blot of *cyclin D1*^*−/−*^ MEFs rescued with MSCV-cyclin D1^WT^-IRES-GFP, MSCV-cyclin D1^C2^-IRES-GFP, or vector control, with antibodies as indicated

In order to determine whether the mutant of cyclin D1 that was defective in binding to G9a was capable of inducing H3K9me2, a comparison was made between the cyclin D1^WT^ and the cyclin D1^C2^ mutant, which is defective in binding G9a (Fig. 1c, e). Transduction of *cyclin D1*^*−/−*^ MEFs with the retroviral vector encoding cyclin D1^WT^ enhanced H3K9me2, whereas transduction with the vector encoding a cyclin D1 mutant defective in binding G9a (cyclin D1^C2^) failed to rescue H3K9me2 (Fig. 3a, b). Western blot analysis of the cyclin D1^WT^ and cyclin D1^C2^ mutant transduced *cyclin D1*^*−/−*^ MEFs for cyclin D1 protein demonstrated the presence of the cyclin D1^WT^ and cyclin D1^C2^ mutant protein (Fig. 3c). The abundance of H3K9me2 was induced greater than twofold by cyclin D1^WT^ but not by cyclin D1^C2^ mutant when normalized for protein loading abundance with Lamin B1 (Fig. 3c).

### H3K9 dimethylation by G9a requires cyclin D1

**Fig. 4:**
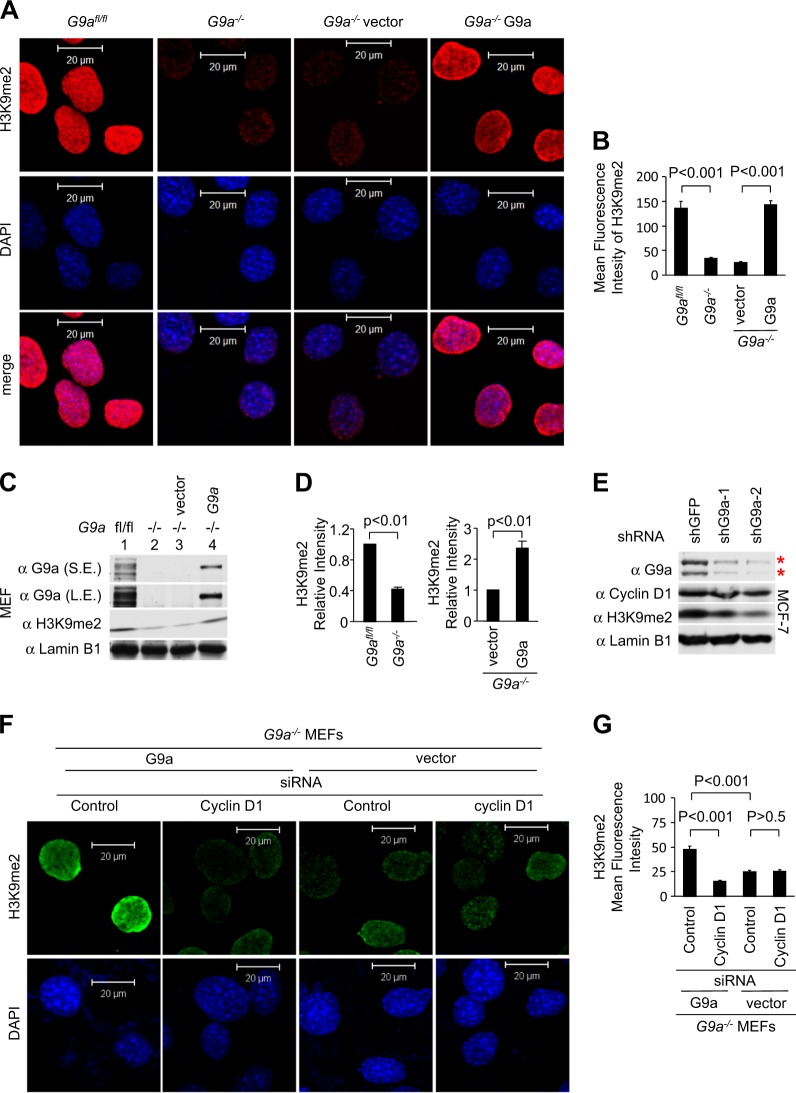
G9a dimethylation of H3K9 requires endogenous cyclin D1. **a**, **b** Confocal microscopy of immunofluorescence for H3K9me2 (red) and nuclear staining with DAPI (blue) in *G9a*^*fl/fl*^ and *G9a*^*−/−*^ mouse embryonic fibroblasts (MEFs), and *G9a*^*−/−*^ MEFs rescued with G9a^WT^ or vector control. Images show the reduction in H3K9me2 in *G9a*^*−/−*^ cells (**a**) and quantitative analysis was shown as mean ± SEM (**b**). **c** Western blot for H3K9me2 and G9a in *G9a*^*fl/fl*^ and *G9a*^*−/−*^ MEFs. The *G9a*^*fl/fl*^, *G9a*^*−/−*^, and *G9a*^*−/−*^ MEFs rescued with G9a and vector control were assessed by western blot for H3K9me2. Lamin B1 was used as a protein loading control. S.E. shorter exposure, L.E. longer exposure. **d** Quantitation of H3K9me2 is shown as mean ± SEM for *N* = 3. **e** MCF-7 cells transduced with two individual shG9a and shGFP control were assessed by western blot for H3K9me2, cyclin D1, and G9a. Lamin B1 was used as a protein loading control. **f**, **g** Confocal microscopy of immunofluorescence for H3K9me2 (green) and nuclear staining with DAPI (blue) in *G9a*^*−/−*^ MEFs rescued with G9a^WT^ or vector control treated with cyclin D1 small interfering RNA. Images show the reduction in H3K9me2 by cyclin D1 siRNA in *G9a*^*−/−*^ plus G9a cells but not in *G9a*^*−/−*^ plus vector cells. Scale bar, 20 μm (**f**) and quantitative analysis was shown as mean ± SEM (**g**)

Given that G9a is crucial for H3K9me2 [5] we confirmed that deficiency of the *G9a* gene in MEFs (*G9a*^*−/−*^) resulted in reduction of H3K9me2 levels compared to the control (*G9a*^*fl/fl*^) cells by immunofluorescence staining (Fig. 4a, b) and western blotting (Fig. 4c, d, *G9a*^*fl/fl*^ vs *G9a*^*−/−*^, *P* < 0.01, S.E., short exposure, L.E., long exposure). Introducing G9a into *G9a*^*−/−*^ MEFs increased H3K9me2 by immunofluorescence compared to the vector control cells (Fig. 4a, b) and by western blot analysis (Fig. 4c, d, *G9a*^*−/−*^ plus vector vs *G9a*^*−/−*^ plus G9a^WT^, *P* < 0.01). Introduction of G9a short hairpin RNA (shRNA) into MCF-7 cells reduced G9a and H3K9me2 levels (Fig. 4e). In order to examine further the requirement for cyclin D1 in G9a function, we conducted cyclin D1 siRNA studies in G9a^−/−^ or *G9a*^*−/−*^ MEF rescued with a G9a expression vector. H3K9me2 was decreased in *G9a*^*−/−*^ cells. Addition of G9a rescued H3K9me2 (Fig. 4f, g). Cyclin D1 small interfering RNA (siRNA) treatment reduced H3K9me2 in *G9a*^*−/−*^ MEF rescued with a G9a expression vector. In *G9a*^*−/−*^ plus vector cells no further decrease in H3K9me2 was observed with cyclin D1 siRNA compared to control siRNA treatment (Fig. 4f, g). Thus, endogenous cyclin D1 is required for the ability of G9a to introduce H3K9me2.

### Cyclin D1 and G9a binding by ChIP-Seq overlap common gene regions

**Fig. 5:**
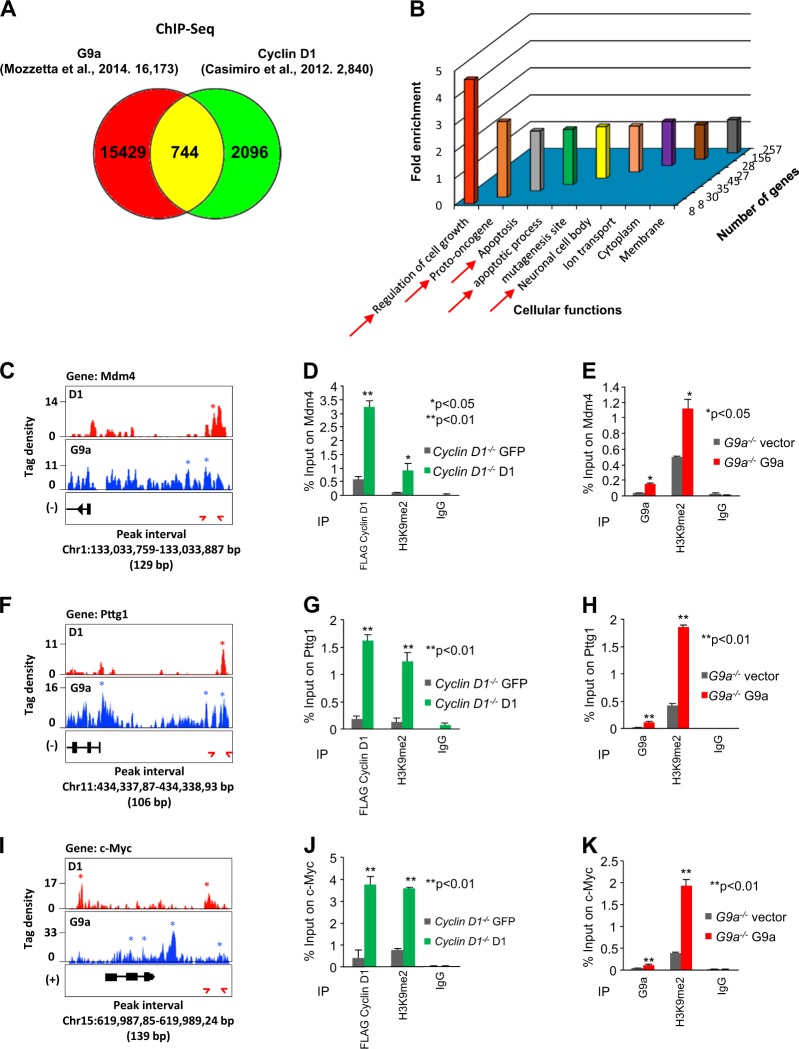
G9a and cyclin D1 bind common regulatory regions of genes in chromatin immunoprecipitation (ChIP)-Seq. **a** Venn diagram depicting the overlapping intervals shared between cyclin D1 ChIP-Seq and G9a ChIP-Seq. **b** Gene Ontology (GO) biological function enrichment scores for overlapping terms for 744 genes common between cyclin D1 ChIP-Seq and G9a ChIP-Seq. **c**–**k** Three genes (*Mdm4*, *Pttg1*, and *Myc*) were selected from the “oncogene” GO biological term and eight genes were selected from the “neuronal activities” GO term (Supplemental Figs. 4,5). **c**, **f**, **i** Depicted are tag density profiles for cyclin D1 intervals (red) and G9a intervals (blue) with respect to the identified genes. Profiles generated by Integrated Genome Browser are depicted for enriched regions binding G9a and the same region of cyclin D1 ChIP-Seq. Enriched intervals are designated by an * for cyclin D1 and a * for G9a. Tag density profiles are not drawn to scale (**c**, **f**, **i**). **d**, **g**, **j** Individual ChIP-qPCR analysis of target genes identified in ChIP-Seq. FLAG (FLAG-cyclin D1) ChIP-qPCR analysis of target genes in *cyclin D1*^*−/−*^ plus GFP vector vs *cyclin D1*^*−/−*^ plus cyclin D1^WT^ rescue mouse embryonic fibroblasts (MEFs), and **e**, **h**, **k** G9a ChIP-qPCR of the same target genes in *G9a*^*−/−*^ plus vector vs *G9a*^*−/−*^ plus G9a^WT^ rescued MEFs. H3K9me2 ChIP-qPCR is conducted in each cell type with IgG as control. Data are shown as mean ± SEM for ChIP-qPCR of FLAG (FLAG-cyclin D1) and H3K9me2 for target genes identified in ChIP-Seq. Significant difference are shown as **P < 0.01 or *P < 0.05 for *cyclin D1*^*−/−*^ plus GFP vector vs *cyclin D1*^*−/−*^ plus cyclin D1^WT^ (**d**, **g**, **j**). Mean ± SEM is shown for ChIP-qPCR of G9a and H3K9me2 for *Mm44*, *Pttg1*, and *c-Myc* genes. Significant difference is shown as **P < 0.01 or *P < 0.05 for *G9a*^*−/−*^ plus vector vs *G9a*^*−/−*^ plus G9a^WT^ (**e**, **h**, **k**)

We next examined occupancy of G9a and cyclin D1 in the genome by comparing ChIP-Seq [30, 33]. G9a binding and cyclin D1 binding, aligned with chromosomal location demonstrated G9a and cyclin D1 were not recruited to the X and Y chromosomes (Supplemental Fig. 3). Sets of genes bound by G9a in mouse embryonic stem cells (mESCs) and cyclin D1 in MEFs were obtained from published ChIP-Seq data [30, 33]. A total of 16,173 G9a-bound genes (GEO database (accession number: GSM1215219)) and 2840 cyclin D1-bound genes were identified (Fig. 5a). Although the data sets were derived from different cell types, 744 genes were identified that were overlapping for both cyclin D1 and G9a binding (Fig. 5a). Thus, 744/16,173 G9a-binding sites are coincident for cyclin D1 (4.6 %) and 744/2840 of the cyclin D1-binding sites are coincident for G9a binding (26.2 %, *P* < 0.01).

The Gene Ontology (GO) functional terms of the genes that bound both cyclin D1 and G9a in ChIP were enriched for genes involved in cellular functions including regulation of cell growth, oncogenesis, apoptosis, and neuronal function (Fig. 5b). From within the overlapping cyclin D1/G9a ChIP-Seq group several genes were chosen for further analysis based on their known role in apoptosis and cellular proliferation (*Mdm4, Pttg1*, and *c-Myc*) (Fig. 5c–k). Neuronal migration and function has been shown to involve cyclin D1 [34, 35] and G9a [36], therefore seven genes were selected from the literature as shown to be linked to neurogenesis through altered expression in mouse models (*Cacna2d4*, *Dlgap3*, *Glra1*, *Scn2a1*, *Kcne2*, *Stx3*, and *Sncb* (Supplemental Figs. 4 and 5)). For the *Pttg1* gene cyclin D1 and G9a ChIP overlapped at a distal site coinciding with a Ctcf site (Fig. 5f–h). The same tag density profiles for ChIP-Seq are shown for cyclin D1 intervals (red) and G9a intervals (blue) in Fig. 5c, f. ChIP–Quantitative Polymerase Chain Reaction (ChIP-qPCR) assays were conducted to verify the ChIP-Seq data of these genes in *cyclin D1*^*−/−*^ cells rescued with FLAG-cyclin D1 or vector control (Fig. 5d, g, j, green bars) and in *G9a*^*−/−*^ cells transduced with wild-type G9a or vector control (Fig. 5e, h, k, red bars). The primers used in ChIP-qPCR and the intervals and peaks in ChIP-Seq are shown in the Supplemental Table 1. Reintroduction of either cyclin D1 or G9a increased H3K9me2 bound to the same regions of these genes (Fig. 5d, e, g, h, j, k, **P < 0.01, *P < 0.05). Reintroduction of cyclin D1 into *cyclin D1*^*−/−*^ cells demonstrated that cyclin D1 was recruited to each target gene examined, accompanied with increased H3K9me2 (Fig. 5d, g, j, Supplemental Fig. 5A, C, E, G, I, K and M). Reintroduction of G9a into *G9a*^*−/−*^ cells resulted in increased G9a and increased H3K9me2 at all target genes demonstrated by ChIP (Fig. 5e, h, k, Supplemental Fig. 5B, D, F, H, J, L and N).

### Cyclin D1 recruits G9a to target genes in the context of chromatin

**Fig. 6:**
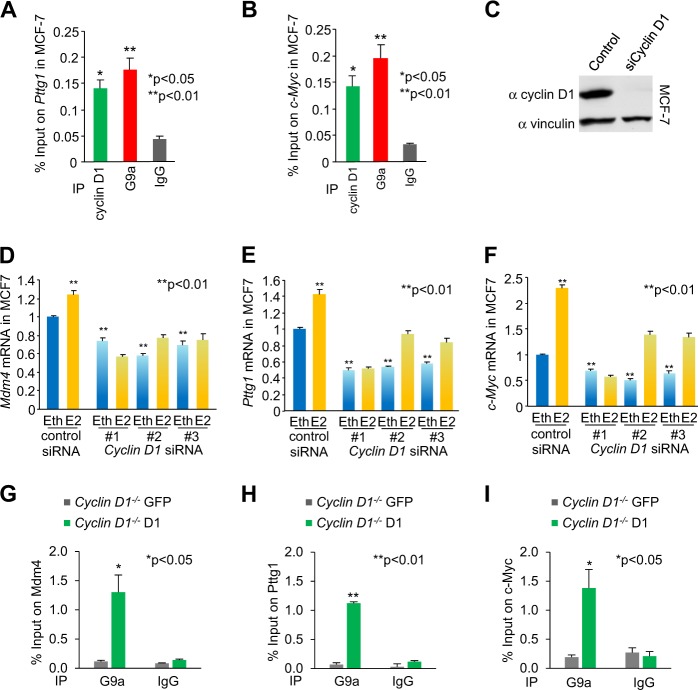
G9a and cyclin D1 bind common regulatory regions of genes in chromatin immunoprecipitation (ChIP)-Seq in MCF-7 cells. **a**, **b** ChIP-qPCR analysis of target genes *Pttg1* and *c-Myc* in MCF-7 cells for G9a and cyclin D1 binding. **c** Western blot for cyclin D1 in cyclin D1 siRNA-treated MCF-7 cells confirmed decrease of cyclin D1 abundance. **d**–**f** Abundance of mRNA for *Mdm4* (**d**), *Pttg1* (**e**), and *c-Myc* (**f**) in MCF-7 with cyclin D1 siRNA or control is shown. Data of qPCR is mean ± SEM for *n* = 3. Eth ethanol, E2 estradiol. **g**–**i** ChIP-qPCR analysis of target genes *Pttg1* and *c-Myc* for G9a binding in cyclin D1 rescue *cyclin* *D1*^*−/−*^ mouse embryonic fibroblasts

The G9a- and cyclin D1-binding regions for *Pttg1* and c-*Myc* were next assessed by ChIP in MCF-7 cells. As shown in MEFs, ChIP enrichment was identified for endogenous cyclin D1 and G9a (Fig. 6a, b). We next assessed the role of endogenous cyclin D1 in regulating the expression of the representative target genes shown to bind G9a and cyclin D1 in ChIP-qPCR and ChIP-Seq. Cyclin D1 siRNA transduction of MCF-7 cells reduced cyclin D1 abundance by western blotting (Fig. 6c), and consistent with a model in which endogenous cyclin D1 maintained expression of the genes identified, cyclin D1 siRNA reduced basal and estradiol-induced expression of the target genes (*Mdm4*, *Pttg1*, and *c-Myc*) (Fig. 6d–f). In order to determine whether cyclin D1 augmented G9a function by enhancing recruitment of G9a to target genes in the context of chromatin, *cyclin D1*^*−/−*^ MEFs were stably transduced with the cyclin D1 expression vector and G9a ChIP was conducted for the target genes (*Mdm4*, *Pttg1*, and *c-Myc*). The reintroduction of cyclin D1 enhanced recruitment of G9a to the target genes *Mdm4* (*n* = 3, *P* = 0.01), *Pttg1* (*n* = 3, *P* < 0.05), and *c-Myc* (*n* = 3, *P* < 0.04) in ChIP assays (Fig. 6g–i).

### Cyclin D1 and G9a are co-located at a subset of LAD-NL contacts

As we had shown that cyclin D1 binds to G9a, we examined the functional significance of this association further. Large genomic regions termed “LADs” interact with the NL. It has been proposed that the LAD/NL interactions constrain the position of chromosomes contributing to the plasticity of chromosome folding [14]. Evidence from photoactivation and photobleaching experiments suggests that during the first two hours of the G_1_ phase of the cell cycle, chromatin is mobile over long distances [37, 38]. G9a methylation of H3 lysine 9 contributes to the anchoring of NL-LAD to laminin [16]. We therefore examined the position of NL regions and compared these locations with the sites of cyclin D1 and G9a binding by ChIP-Seq (Supplemental Figure 6). NL regions were selected based on prior publications of NL in ESC (orange bars) and in MEFs (violet-/gray-colored bars) [39, 40]. There are approximately 1189 LAD in MEFs [41]. There were 100 sites in which coincident binding of cyclin D1 and G9a occurred. Furthermore, the coincident binding of cyclin D1 and G9a was located at the edges of the LAD. We found 31 cyclin D1- and G9a-binding genes to be located on the LAD edges by ChIP-Seq (*P* < 0.001). The 31 cyclin D1- and G9a-binding genes were located near the edges site within a distance of +500 bp (*P* < 0.001). No binding of cyclin D1 and G9a was identified with any of the approximately 1800 genes located on the X chromosome.

**Fig. 7:**
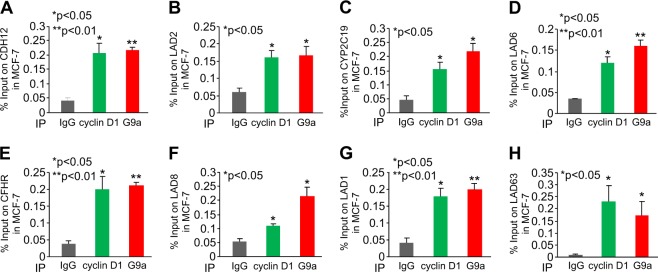
G9a and cyclin D1 bind common regions of Lamina-associated genes in chromatin immunoprecipitation (ChIP). Individual ChIP-qPCR analysis of Lamina-associated target genes in MCF-7 cells. Cyclin D1 and G9a ChIP-qPCR analysis of target is shown as mean ± SEM for *n* = 3. **a** CDH12, **b** LAD2, **c** CYP2C19, **d** LAD6, **e** CFHR, **f** LAD8, **g** LAD1, and **h** LAD63

In prior studies a subset of genes was shown to have G9a recruitment to their regulatory regions (*CDH12*, *CFHR*, *CYP2C19*, and *LAD1*,*2*,*6*,*8*,*63*). Interestingly, cyclin D1- and G9a-binding at these regions were shown to coincide with the NL. We therefore assessed the binding of G9a to several of these gene regions as previously described [14]. Consistent with prior studies, G9a associated in ChIP at the region of genes previously identified at the NL, and shown to be modified by H3K9me2 in a G9a-dependent manner (Fig. 7a-h). Furthermore, we demonstrated that cyclin D1 was recruited to these same regions in Lamina-associated genes by ChIP-qPCR (Fig. 7a–h, cyclin D1 ChIP are shown as green bars).

### Cyclin D1 binding to G9a determines the interaction of LADs with NL

**Fig. 8:**
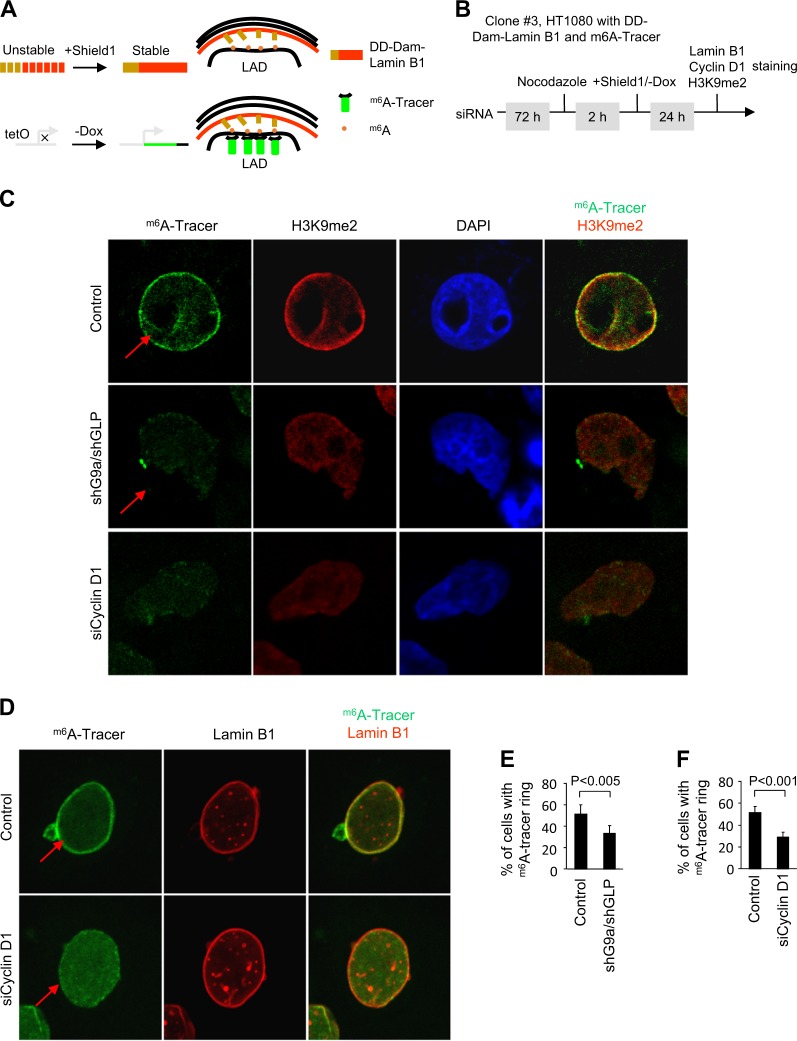
Cyclin D1 determines accumulation of G9a-mediated nuclear lamina interactions at the nuclear periphery. **a** Illustration of the inducible ^m6^A-tracer/Dam-lamin B1 system in the HT1080 cell line stably expressing the Shield1-inducible Dam-Lamin B1 and the Tet-off ^m6^A -tracer (known as the line clone 3). Inducible Dam-Lamin B1 expression was established by the fusion of a destabilization domain (DD), which causes Dam-Lamin B1 to be rapidly targeted for proteasomal degradation unless the protein is shielded by the synthetic small molecule Shield1.The induction of ^m6^A-tracer is based on the Tet-Off system, where the removal of doxycycline (Dox) results in the activation of transcription. **b** Schematic representation of the experimental protocol. **c** Confocal microscopy images showing both G9a shRNA/GLP shRNA and cyclin D1 siRNA decreases H3K9me2 staining (red) in H1080 cells. **d** Representative confocal microscopy images showing cyclin D1 siRNA decreases accumulation of ^m6^A-tracer signal (green) at the nuclear periphery after induction of Dam-Lamin B1. Lamin B1 staining is shown in red. **e** Quantitative analysis of ^m6^A-tracer incorporation at the nuclear periphery after induction of Dam-Lamin B1 expression shown as % of cells incorporating tracer for *n* > 200 separate cells for shG9a/shGLP treatment and its vector control. Data are percentage ± 95% confidence interval (CI). **f** Quantitative analysis of ^m6^A-tracer incorporation at the nuclear periphery shown as % of cells incorporating tracer for *n* > 400 separate cells cyclin D1 siRNA treatment as well as its control. Data are percentage ± 95% CI

The stochastic interaction of NL with LADs was associated with increased H3K9me2 status [14]. Although G9a methylation of H3K9 is important for the anchoring of NL-LAD to laminin, the mechanisms governing the LAD-NL interaction are poorly understood. The ^m6^A (adenine-6-methylation) tracer technology was developed to follow genomic-NL interactions at a single-cell level [14]. In this approach, adenine-6-methylation (^m6^A) is used to in vivo tag genomic regions in contact with nuclear proteins. In order to determine the role of cyclin D1 in the G9a-regulated NL/LAD function [14], we deployed an HT1080-derived clone in which the Dam-Lamin B and the ^m6^A-tracer can be independently induced (Fig. 8a). In order to determine the role of cyclin D1, siRNA to cyclin D1 was deployed. After 72 h of cyclin D1 siRNA treatment, the ^m6^A-tracer was induced upon removal of doxycycline using a tet-off system (Fig. 8b). The inducible Dam-Lamin B1 expression is dependent upon the fusion of a destabilization domain (DD), which ensures that Dam-Lamin B1 is rapidly targeted for proteasomal degradation in the absence of the small molecule called Shield1 [42].

In previous studies, neither Dam nor the fusion of DD affected the genome NL interactions [14]. The ^m6^A-tracer was identified in the nuclear periphery 24 h after induction of Dam-Lamin B1 expression. The 24 h time point was used, because of the high resolution of ^m6^A-tracer Lamin B1 interaction at that time point (Fig. 8b) [14]. The immunofluorescent labeling showed that the ^m6^A-tracer in green, after induction of Dam-Lamin B1, co-localizes with H3K9me2 in red (Fig. 8c in yellow). In contrast, shG9a/shGLP-transduced cells, and cyclin D1 siRNA-transduced cells, showed reduced ^m6^A-tracer at the nuclear periphery and reduced co-localization with H3K9me2 (Fig. 8c). Figure 8d shows the location of Lamin B1 at the nuclear periphery and the merged image of the ^m6^A-tracer and Lamin B1 evidenced in control siRNA-treated cells (Fig. 8d, upper panel). Cyclin D1 siRNA decreased accumulation of ^m6^A-tracer at the nuclear periphery (Fig. 8d). shG9a/shGLP reduced the percentage of cells with the ^m6^A-tracer ring by >40% (Fig. 8e). Quantitative analysis of *n* = 415 cells demonstrated a 50% reduction in the percentage of cells with ^m6^A-tracer in cyclin D1 siRNA-treated cells (Fig. 8f, *P* < 0001). Together, these studies demonstrate that endogenous cyclin D1, like endogenous G9a, promotes H3K9 dimethylation and LAD-NL interaction.

### Cyclin D1 and G9a are overexpressed in ERα^+^ breast cancer

G9a is overexpressed in many types of cancer [43–45] and has been shown to augment tumorigenesis [46, 47], reviewed in [48]. Similarly, cyclin D1 overexpression augments breast cancer in transgenic mice [49, 50]. We investigated whether cyclin D1 and G9a expression was increased in human breast cancer and whether cyclin D1 correlated with increased G9a abundance in particular breast cancer subtypes. The relative abundance of cyclin D1 and G9a was correlated in normal breast epithelium (*P* = 0.003, Supplemental Fig. 7A and B). Consistent with prior studies, cyclin D1 mRNA levels were increased in breast cancer compared with healthy breast tissue (Supplemental Fig. 7C). G9a was also increased in human breast cancer compared with healthy breast tissue (Supplemental Fig. 7D). G9a mRNA levels correlated with cyclin D1 mRNA levels in healthy breast tissue (*R* = 0.29, *P* = 0.003) and ERα^+^ breast cancer samples (*R* = 0.27, *P* = 8.07e-30). But no significant correlation was observed in ERα^−^ breast cancer patients (Supplemental Fig. 7E and F). Increased expression of either cyclin D1 or G9a was not significantly correlated with poor outcome breast cancer (Supplemental Fig. 7G and H).

Together these data demonstrate that cyclin D1 binds and recruits G9a to induce H3K9 dimethylation, which is known to promote NL-LAD interactions at the nuclear periphery (Supplemental Fig. 8). Cyclin D1 recruits G9a, which promotes H3K9me2 at the promoter of target genes, which may thereby regulate gene expression and signaling.

## Discussion

The current studies define novel functions for cyclin D1 determined through binding of the protein methyltransferase G9a. First, using G9a rescued *G9a*^*−/−*^ MEFs, we showed that endogenous cyclin D1 was required for G9a to induce H3K9me2. Second, in genome-wide analysis, 26.2% of cyclin D1-binding sites were found to be coincident for G9a binding (*P* < 0.01). Furthermore, there was enrichment for cyclin D1 and G9a binding at the edges of LADs. Third, we showed endogenous cyclin D1 augments recruitment of G9a to target genes in ChIP (*Pttg1* and *Mdm4*). Fourth, using an adenine-6-methylation tracer, we demonstrated the requirement for endogenous cyclin D1 in maintaining the G9a function of H3K9me2 visualizing incorporation at NL-LAD sites. Collectively, these studies demonstrate a novel chaperone function for cyclin D1 to recruit G9a and thereby augment H3K9 dimethylation.

Analysis of cyclin D1 and G9a ChIP-Seq demonstrated a significant overlap in binding to common genes. The GO terms corresponding to the genes bound by both cyclin D1 and G9a included regulation of cell growth, apoptosis, and neural function. G9a is known to be overexpressed in a variety of malignancies [43–45] and enhances tumorigenesis [46, 47]. Similarly, cyclin D1 is overexpressed in malignancies and enhances mammary tumorigenesis [49, 50]. Cyclin D1 promotes neurite extension [34, 35] and using anti-sense, cyclin D1 was shown to be essential for Nerve Growth Factor (NGF)-induced neurite extension [34]. Cyclin D1 and G9a are expressed in the developing central nervous system [51] and participate in neurogenesis [36], therefore an additional seven genes were selected from the neurogenesis function. At each of the genes assessed, cyclin D1 augmented H3K9me2 at the target gene in chromatin. Furthermore, cyclin D1 was shown to augment G9a recruitment to target genes. Mutational analysis herein demonstrated the binding and augmentation of G9a KMT function was independent of the cyclin D1 kinase function, consistent with recent studies that showed cyclin D1 induced mammary tumorigenesis independently of its kinase function [30, 50]. The mechanisms by which cyclin D1 governs neurogenesis is not known, however based on genetic deletion studies in the mouse, may involve the Notch pathway [52]. Cyclin D1 activates Notch signaling by enhancing γ secretase activity [53], and the neural stem cell-promoting function of cyclin D1 is distinct from its cell cycle function [54]. Whether the augmentation of G9a function by cyclin D1 contributes to tumorigenesis and neurogenesis remains to be determined.

The regulation of gene expression and the maintenance of genomic integrity is influenced by interphase chromosomes architecture. The mechanisms governing chromosomal positioning and architecture is poorly understood. The NL interacts with multiple LAD, which cover 35–40% of the mammalian genome, thereby restraining the position of chromosomes. The functional significance of the interaction between cyclin D1 and G9a included the requirement for endogenous cyclin D1 to maintain the G9a-dependent genome-NL interactions [14]. As the abundance of cyclin D1 is regulated during the cell cycle and by diverse oncogenic and mitogenic signals [23], the current studies provide a potential mechanism by which the multiple dynamic inputs that govern cyclin D1 abundance may, in turn, influence chromosomal architecture.

The current studies demonstrate cyclin D1 functions to recruit G9a and thereby induce dimethylation of H3K9 as shown by immunofluorescence and western blotting. Furthermore, cyclin D1, recruited in the context of chromatin, induced H3K9me2. Several distinct enzyme complexes are either recruited by cyclin D1^WT^ in the context of chromatin including SUV39H1, HP1α, HDAC, and p300 [26, 29], or in the case of a cyclin D1 synthetic mutant (T286A), associates with PRMT5 [55]. G9a has an ability to methylate additional non-histone substrates, and it will be of interest to determine whether cyclin D1 is capable of augmenting these additional G9a-mediated functions. H3K9me2, which is an abundant histone mark maintained by several enzymes in addition to G9a (SETDB1 and SUV39H1), was maintained by endogenous cyclin D1. Whether G9a is the only H3K9 dimethylase bound by cyclin D1 remains to be determined. Further analysis of the genes targeted by G9a and cyclin D1 that govern chromosomal segregation and oncogenesis may provide important mechanistic insights into these fundamental processes.

## Materials and methods

### Plasmids

The pBIND plasmids expressing GAL4-tagged cyclin D1 wild type and mutations were as described previously [56]. The pcDNA3.1 plasmids encoding FLAG-tagged G9a^WT^ and deletion mutations were obtained from Dr. Eiji Hara (Cancer Institute, Japanese Foundation for Cancer Research, Tokyo, Japan) [57]. The pLKO lentiviral plasmid vectors of human G9a shRNA and GLP shRNA were purchased from Sigma.

### Cell culture and reagents

MCF-7 and HEK 293T cell lines were from the American Type Culture Collection (ATCC, Manassas, VA). *Cyclin D1*^*+/+*^ MEFs, *cyclin D1*^*−*/−^ MEFs, *cyclin D1*^*−/−*^ MEFs rescued with cyclin D1^WT^, cyclin D1^KE^ mutant, or vector control were as described before [58]. The *G9a*^*fl/fl*^ MEFs and *G9a*^*−/−*^ MEFs were a generous gift from Dr. Alexander Tarakhovsky (The Rockefeller University, New York) [59]. The HT1080 cell line stably expressing the Shield1-inducible Dam-Lamin B1 and the Tet-off ^m6^A -tracer (known as the line clone 3) was provided by Dr. Bas van Steensel (Netherland Cancer Institute, Amsterdam, Netherland) [14]. The restriction endonuclease *Dpn*I cuts the sequence G^m6^ATC (^m6^A is short for adenine-6-methylation) but not GATC. The truncation mutation, a C-terminal fragment of 109 amino acids of *Dpn*I fused to enhanced green fluorescent protein (EGFP) is referred as ^m6^A-tracer. The ^m6^A-tracer signal is reduced beyond detection (EGFP detected using microscopy) after 24 h incubation with 50 ng/ml doxycycline (Research Products International Corp. Mount Prospect, IL). Cells were cultured in Dulbecco’s modified Eagle’s medium (DMEM) containing streptomycin and penicillin (100 mg of each/liter) and 10% fetal bovine serum (FBS). To promote stability of long-term culture, the clone 3 of HT1080 cells were maintained in 400 μg/ml G418 (Santa Cruz Biotechnology) and 2 μg/ml puromycin (Sigma).

MCF-7 and HEK 293T were recently authenticated by ATCC. The MEFs generated by the Pestell lab were authenticated by IDEXX Bioresearch. All of the cells were tested for Mycoplama contamination using the ATCC Universal Mycoplasma Detection Kit.

### siRNA knockdown of endogenous cyclin D1

The siRNAs specifically targeting human cyclin D1 mRNA (Hs_CCND_1, Hs_CCND_2, and Hs_CCND_3), purchased from Qiagen, were used for suppressing endogenous cyclin D1 expression in HT1080 cells. For suppression of endogenous cyclin D1 expression in *G9a*^*−/−*^ MEFs rescued with wild-type G9a or vector control, mouse Ccnd1_2 FlexiTube siRNAs (Catalog #SI00943642) specifically targeting mouse cyclin D1 mRNA were also purchased from Qiagen. The cyclin D1 siRNAs or negative control siRNA (Qiagen) were transfected into the cells with the Lipofectamine 2000 (Invitrogen, Carlsbad, CA) according to the manufacturer’s instruction. For HT1080 cells, which stably expressing the Shield1-inducible Dam-Lamin B1 and the Tet-off ^m6^A-tracer, after transfection, the cells were sequentially treated with 10 μM Nocodazole (Sigma) for 72 h and 0.5 μM Shield1 (Clontech) in DMEM with 10% tetracycline-free FBS for 2 h to induce Dam-Lamin B1 and ^m6^A-tracer expressing. The immunofluorescence staining was performed another 24 h later. For *G9a*^*−/−*^ MEFs, immunofluorescence staining was processed 72 h later after siRNA transfection.

### Immunofluorescence staining

Immunofluorescence staining was conducted as described previously [56]. The cells in four-well chamber slides were fixed for 10 min at room temperature (RT) with 4% paraformaldehyde, and subsequently with cold methanol at −20 °C for 5 min. The slides were then treated with 0.2% Triton X-100 for 5 min at RT and blocked with 2% bovine serum albumin overnight at 4 °C. For H3K9me2 and cyclin D1, G9a and cyclin D1 co-staining in MEFs the primary antibodies used were mouse monoclonal anti-H3K9me2 (ab1220) (Abcam Inc.) (1/800), mouse monoclonal anti-G9a (clonal A8620A) (PP-A8620A-00) (R&D System) (1/500), and rabbit polyclonal anti-cyclin D1 (clone H-295) (sc-753) (Santa Cruz Biotechnology, Santa Cruz, CA) (1/200). The secondary antibodies used were Alexa Fluor 568-conjugated goat anti-mouse immunoglobulin G (IgG) (Molecular Probes, Inc.) (1/500) and Alexa Fluor 647-conjugated F(ab′)2 fragment of goat anti-rabbit IgG (Molecular Probes, Inc.) (1/500). For HT1080 immunostaining the primary antibodies used were mouse monoclonal anti-cyclin D1 (clone DCS-6) (sc-20044) (Santa Cruz Biotechnology, Santa Cruz, CA) (1/200), rabbit polyclonal anti-Lamin B1 (ab16048) (Abcam Inc.) (1/900), and mouse monoclonal anti-H3K9me2 (ab1220) (Abcam Inc.) (1/800). The secondary antibodies used were rhodamine-conjugated F(ab′)2 fragment of goat anti-rabbit IgG (Jackson Immuno Research Laboratories, Inc.) (1/500) and Alexa Fluor 633-conjugated F(ab′)2 fragment of goat anti-mouse IgG (Molecular Probes, Inc.) (1/500). The samples were visualized on a Zeiss LSM 510 META Confocal Microscope with a ×63 objective.

### Immunoprecipitation and western blotting

HEK 293T cells was co-transfected with pBIND-FLAG-G9a and pcDNA3.1-GAL4-cyclin D1. The transfected cells were lysed 48 h later in IP buffer (10 mM Tris-HCl at pH 7.4, 150 mM NaCl, 1 mM EDTA, 1 mM EGTA, 1% Triton X-100, 0.5% IGEPAL CA-630, 10% glycerol, 0.2 mM sodium orthovanadate, 0.1 mM phenylmethylsulfonyl fluoride, 10 μg/ml aprotinin, 1 μg/ml leupeptin, and 1 μg/ml pepstatin). For each IP, 1 ml cell lysate containing 1 mg protein was incubated overnight with 10 μl anti-FLAG M2-agarose beads (A2220, Sigma-Aldrich) at 4 °C. The immunoprecipitates were washed five times with IP buffer, and lysed in 20 μl of ×2 sample buffer. The immunoprecipitates and the corresponding lysates containing 50 μg protein were analyzed by western blotting as previously described [58]. Antibodies that were used for western blotting included: mouse anti-FLAG antibody (M2, F-3165, Sigma-Aldrich), anti-vinculin antibody (V9131, Sigma-Aldrich), mouse anti-cyclin D1 antibody (DCS-6, SC-20044, Santa Cruz), rabbit anti-Lamin B1 (ab16048, Abcam), mouse anti-G9a (clonal A8620A) (PP-A8620A-00) (R&D System), mouse anti-H3K9me2 (ab1220, Abcam), and rabbit anti-G9a antibody (Catalog # 07–551, Millipore).

### ChIP assay

ChIP analysis was performed using Magna ChIP kits (Millipore) according to the manufacturer’s instruction. The following antibodies were used in ChIP: mouse monoclonal anti-FLAG (M2) (F-3165, Sigma-Aldrich) (for FLAG-cyclin D1), mouse anti-G9a (clonal A8620A) (PP-A8620A-00) (R&D System), rabbit anti-G9a (clonal C6H3) (Cell signaling), and mouse anti-H3K9me2 (ab1220) (Abcam Inc.). For a negative control, mouse IgG was from the Magna ChIP kits (Millipore). SYBR Green PCR kit (Invitrogen) was used in the ChIP-real time PCR (ChIP-qPCR). The primers used in ChIP-qPCR are shown in the Supplemental Table 1.

### Generation of cyclin D1 knockout mice and immunohistochemistry staining

The Animal protocol used in this study was approved by the Institutional Animal Care & Use Committee at Thomas Jefferson University. C57BL/6J *cyclin D1*^*fl/fl*^ mice were a kind gift from Dr. Piotr Sicinski (Dana-Farber Cancer Institute, Boston, MA). C57BL/6J Rosa26-CreERT2 mice, which express the tamoxifen-inducible CreERT2 fusion protein were from Dr. Thomas Ludwig (Columbia University, New York, NY). *Cyclin D1*^*fl/fl*^*-Rosa26*^*CreERT2/CreERT2*^ mice were generated by crossing *cyclin D1*^*fl/fl*^ mice with Rose26-Cre-ERT2 mice. Cyclin D1 knockout mice were generated with *Cyclin D1*^*fl/fl*^*-Rosa26*^*CreERT2/CreERT2*^ mice by intraperitoneal injection of tamoxifen (1 mg/200 µl sunflower seed oil) for 5 days. *Cyclin D1*^*wt/wt*^*-Rosa26*^*CreERT2/CreERT2*^ mice were used as control. Mammary glands from these mice were collected 4 weeks after IP injection of tamoxifen. Immunohistochemistry staining was conducted on paraffin-embedded mammary gland sections. The primary antibodies used were mouse monoclonal anti-cyclin D1 (clone DCS-6) (sc-20044) (Santa Cruz Biotechnology, Santa Cruz, CA), rabbit polyclonal anti-G9a (clone C6H3) (Cell Signaling Technology, Inc, Danvers, MA), and mouse monoclonal anti-H3K9me2 (ab1220) (Abcam Inc.).

### Cyclin D1- and G9a-bound regions by ChIP-Seq and related biological function pathway analysis

Genomic DNA regions bound by cyclin D1 [30] were compared to those bound by G9a as determined via ChIP-Seq analysis [33]. We retrieved genomic locations of G9a peaks from the GEO database (accession number GSM1215219). Cyclin D1 peaks were identified using MACS v1.4 software with the following parameters: –shiftsize 50 –nomodel [60]. We identified 6834 cyclin D1 peaks at the false discovery rate cutoff of 5%. The average size of a G9a-enriched region (peak) is 800 bp. The average size of a cyclin D1-enriched region (peak) is 400 bp. A cyclin D1 and G9a peaks were deemed to be co-localized at a locus when the enriched regions shared overlapping nucleotide binding. Using this criterion, we found that 755 of cyclin D1 peaks overlap G9a peaks genome-wide. In all, 744 genes corresponding with these 755 peaks were located within 25 kb of gene transcription start sites [61–63]. The common genes between the two sets were queried for biological function using the PANTHER classification system [64, 65] of GO terms within **D**atabase for **A**nnotation, **V**isualization and **I**ntegrated **D**iscovery (DAVID) [66, 67]. GO-enrichment analysis was conducted using PANTHER (Protein ANalysis THrough Evolutionary Relationship). The PANTHER Classification System of GO terms involved in a Biological Process was used to cluster genes based on function. The enrichment score was based on the EASE scores; a modified Fisher exact *P*-value for gene enrichment analysis. The following parameters within DAVID were kept at default; EASE threshold = 0.1 and count threshold = 2. PANTHER pathways (9 total) were chosen based on Fisher exact *P*-value > 0.05. Select genes were further analyzed using the Integrated Genome browser (Affymetrix) to depict the cyclin D1 and G9a tracks [66, 67].

### **A**nalysis of G9a- and cyclin D1 DNA-bound regions at chromosomal locations and to LAD

A list of genes was generated for G9a- and cyclin D1-bound genomic locations based on prior published ChIP-Seq data [30, 33, 41]. A sequentially expanding list of nearest-neighbor genes was compared between G9a and cyclin D1 ChIP-Seq data by making iterative expansions of the associated interval (500 bp, 1 kb, 2 kb, 5 kb, and 10 kb). We obtained the list of G9a associated Ensembl gene IDs from Mozzetta et al. [33], supplementary table (http://www.cell.com/cms/attachment/2038986201/2052742122/mmc2.xlsx), column E (G9a ChIP-Seq in TT2 mESC). The list of cyclin D1-associated genes was based on overlapping the cyclin D1 interval coordinate data to the NCBI protein-coding genes [30]. Genes found under cyclin D1 intervals were validated by comparing the cyclin D1 gene sets obtained by two different researchers using two different methods. Both methods returned the same core genes, with the newer method returning more genes (478). Both methods found the overlap of cyclin D1/G9a genes statistically significant. The first method was the NCBI overlap method just described. The second method found Ensembl genes under or within 10 kb of the cyclin D1 intervals using the Ensembl Release 67 from May 2012 because the cyclin D1 interval data were mapped onto the older assembly. The additional genes from the second method were likely found because there was no loss from a NCBI-to-Ensemble gene ID translation when comparing G9a Ensembl IDs and cyclin D1 NCBI vs Ensembl gene IDs. To obtain the list of genes that possess both overlapping genes of cyclin D1 and G9a, we analyzed the intersection of G9a and cyclin D1 gene sets. Statistical significance of the overlap was conducted using hypergeometric test [68, 69]. The intersecting cyclin D1 and G9a protein-coding genes were mapped to prior published LAD coordinates for mESCs and fibroblasts [70, 71].

### Statistical analysis

The statistical significance of mean difference was determined with two-tailed Student’s *t*-test. The statistical significance of two sample proportions was determined with two-tailed two-sample *z*-test.

## Disclaimer

The Pennsylvania Department of Health specifically disclaims responsibility for analyses, interpretations, or conclusions

## Supplementary information

Supplementa Table 1

Supplemental Figure Legends

Supplemental Figures
