# Supplementary material for: Cyclin D1 integrates G9a-mediated histone methylation
Source: Oncogene. 2019 Feb 4;38(22):4232–49. doi: 10.1038/s41388-019-0723-8 (PMC6542714; doi:10.1038/s41388-019-0723-8)
Supplement: Supplementary file 1 — Supplementa Table 1 [file 41388_2019_723_MOESM1_ESM.docx]

| **Gene** | **Forward sequence** | **Reverse sequence** | **Amplicon bp** | **Chr.** | **5' Coordinates** | **3' Coordinates** |
| --- | --- | --- | --- | --- | --- | --- |
| Myc | CCTGCTGCCTCTCTACAACC | CCAGGAGGACAGCAAGTCAT | 139 | 15 | 61998785 | 61998924 |
| Mdm4 | CCGCGTCGCTCTAAGTTTAC | TCGTCTTCCCGTGAGAGATT | 129 | 1 | 133033759 | 133033887 |
| Pttg1 | CGGATGGTTTTGAGTGGTCT | AGGACTCACTGCAGGGGATA | 106 | 11 | 43433787 | 43433893 |
| Cacna2d4 | CTCTTCTTGGCAGCTTGGAA | GACAGGCCCACAGCTTAGAT | 126 | 6 | 119329335 | 119329461 |
| Dlgap3 | TTCTTGCTGTCTGCGCTTT | TCAGTACCCGGGACAGCTC | 121 | 4 | 127173611 | 127173732 |
| Kcne2 | ACTGCGCCTGCGTATCAAG | GCTTGCTGCTCACAAACCAC | 119 | 16 | 92301202 | 92301321 |
| Glra1 | TAGAGGGACGAGGTGTCAGA | TCCAGCGAGTAGAGCTTCAG | 149 | 11 | 55607858 | 55608007 |
| Stx3 | GCGGAAACTTGAAACACCTC | GTCATCCTGTCCCACCTTTT | 133 | 19 | 11770237 | 11770370 |
| Sncb | GGAATTTATGTCGGAGAGCA | CTCTTGGGCAGTGGAAAAAC | 110 | 13 | 54750851 | 54750961 |
| Scn2a1 | GCCTGTTCTTACTTCAGTTACCAG | GAAGCAAGAAGACTGCAGCTAAA | 133 | 2 | 65668642 | 65668775 |

**Supplemental Table 1. ChIP-qPCR primers**
