# Supplementary material for: Cyclin D1 integrates G9a-mediated histone methylation
Source: Oncogene. 2019 Feb 4;38(22):4232–49. doi: 10.1038/s41388-019-0723-8 (PMC6542714; doi:10.1038/s41388-019-0723-8)
Supplement: Supplementary file 2 — Supplemental Figure Legends [file 41388_2019_723_MOESM2_ESM.docx]

CYCLIN D1 INTEGRATES G9a-MEDIATED HISTONE METHYLATION

**Zhiping Li, Xuanmao Jiao, Gabriele Di Santi, Adam Ertel, Mathew C. Casimiro, Min Wang, Sanjay Katiyar, Xiaoming Ju, D.V. Klopfenstein, Aydin Tozeren, Iouri Chepelev,** **Albert Jeltsch, Richard G. Pestell**

**SUPPLEMENTAL FIGURE LEGENDS:**

**Supplemental Figure 1. Cyclin D1 induces H3K9me2 and is independent of cdk-binding function.** Confocal microscopy of immunofluorescence for cyclin D1 (far red), nuclear staining with DAPI (blue), and H3K9me2 (red) in *cyclin D1^-/-^* MEFs rescued with MSCV-cyclin D1^WT^ (wild type), MSCV-cyclin D1^KE^ (KE mutant is defective in cdk binding), or vector control. Scale bar, 20 μm. H3K9me2 staining is restored in *cyclin D1^-/-^* MEF with either MSCV-cyclin D1^WT^ (wild type), MSCV-cyclin D1^KE^.

**Supplemental Figure 2. Conformation of cyclin D1 deletion in *cyclin D1* conditional knock-out mouse mammary gland epithelial cells**. (A) Genomic analysis of the transgenic mice for the cyclin D1 floxed allel, (B) for the presence of the mTmG fusion allele and (C), homozygous Cre-ErT2 as described in the Materials and Methods. (D) Immunohistochemistry staining of cyclin D1 on paraffin embedded mammary gland sections in *cyclin D1^wt/wt^-Rosa26^CreERT2/CreERT2^* mice and c*yclin D1^fl/fl^-Rosa26^CreERT2/CreERT2^* mice treated with tamoxifen with quantitation shown as mean ± SEM.

**Supplemental Figure 3. Comparative analysis of G9a and cyclin D1 DNA bound regions.** Mouse genomic map of cyclin D1 and G9a Chip-Seq intervals relative to protein coding genes.

**Supplemental Figure 4. Chromosomal alignment of Cyclin D1 ChIP Seq and G9a ChIP Seq.** Depicted are tag density profiles for cyclin D1 intervals (red) and G9a intervals (blue) with respect to the identified genes. Profiles generated by Integrated Genome Browser are depicted for enriched regions binding G9a and the same region of cyclin D1 ChIP-Seq. Enriched intervals are designated by an * for cyclin D1 and a * for G9a. Tag density profiles are not drawn to scale. The genes that bound both cyclin D1 and G9a in ChIP-Seq were identified within the gene-ontology term target of neuronal differentiation. Individual analysis of target genes is shown for ChIP-Seq. (A) CaCna2d4, (B) Kcne2, (C) Dlgap3, (D) Stx3, (E) Glra1, (F) Sncb and (G) Scn2a1.

**Supplemental Figure 5. Genomic co-localization of Cyclin D1 and G9a ChIP-qPCR.** (A, C, E, G, I, K, and M) FLAG (FLAG-Cyclin D1) ChIP-qPCR analysis of the neuronal differentiation target genes in *cyclin D1^-/-^* plus GFP vector vs *cyclin D1^-/-^* plus cyclin D1^WT^ rescue MEFs, and (B, D, F, H, J, L and N) G9a ChIP-qPCR of the same target genes in *G9a^-/-^* plus vector vs *G9a^-/-^* plus G9a^WT^ rescue MEFs. H3K9me2 ChIP-qPCR is conducted in each cell type with IgG as control. Data is shown as mean ± SEM for ChIP-qPCR of FLAG (FLAG-cyclin D1) and H3K9me2 for target genes identified in ChIP-Seq including *Cacna2d4* (A, B), *Kcne2* (C, D), *Dlgap3* (E, F), *Stx3* (G, H), *Glra1* (I, J), *Sncb* (K, L) and *Scn2a1* (M, N) genes. Significant difference are shown as ** P<0.01 or * P<0.05 for *cyclin D1^-/-^* plus GFP vector vs *cyclin D1^-/-^* plus cyclin D1^WT^ (A, C, E, G, I, K and M) or for *G9a^-/-^* plus vector vs *G9a^-/-^* plus G9a^WT^ (B, D, F, H, J, L and N).

**Supplemental Figure 6.** The plotted LAD are for ESC and for MEF regions on the mouse genome with 478 overlapping genes of Cyclin D1 by ChIP Seq and G9a ChIP Seq. The KEY for each chromosome, which contains 5 rows of visual information per chromosome: Row 1: Top narrow pink bars: G9a/cyclinD1 ChIP-Seq overlap genes on forward strand. Row 2: Next narrow pink bars: G9a/cyclinD1 ChIP-Seq overlap genes on reverse strand. Row 3: Orange wider bars: LAD ESC. Row 4: Violet wider bars: LAD MEF. Row 5: Bottom bar: Cytoband line.

**Supplemental Figure 7. Cyclin D1 and G9a are overexpressed in ERα^+^ breast cancer correlating with poor outcome.** (A and B) Cyclin D1 transcript level plotted versus average G9a expression level revealed correlation between G9a and cyclin D1 in normal breast tissue (P < 0.003). (C and D) Whisker plot of cyclin D1 (C) and G9a (D) expression by ERα status derived from combined breast cancer microarray datasets that were assigned to Healthy (normal breast), All BC (breast cancer), ERα^+^ BC and ERα^-^ BC. (E and F) Relative expression levels and correlations of cyclin D1 and G9a for either healthy, all breast cancer (E), and ERα^+^ vs ERα^-^ breast cancer samples (F). (G and H) Kaplan-Meier plot showing differences in overall survival for cyclin D1 (G) or G9a (H) in ERα^+^ breast cancer. (N=358).

**Supplemental Figure 8. Cyclin D1 recruits G9a to augment H3K9me2 at nuclear lamina**. Schematic representation of the mechanism by which cyclin D1 binding to G9a augments recruitment of G9a to transcriptional sites and to lamina associated domains (LAD) to thereby augment H3K9me2.
