# Supplementary material for: Cyclin D1 integrates G9a-mediated histone methylation
Source: Oncogene. 2019 Feb 4;38(22):4232–49. doi: 10.1038/s41388-019-0723-8 (PMC6542714; doi:10.1038/s41388-019-0723-8)
Supplement: Supplementary file 3 — Supplemental Figures [file 41388_2019_723_MOESM3_ESM.pptx]

## Slide 1
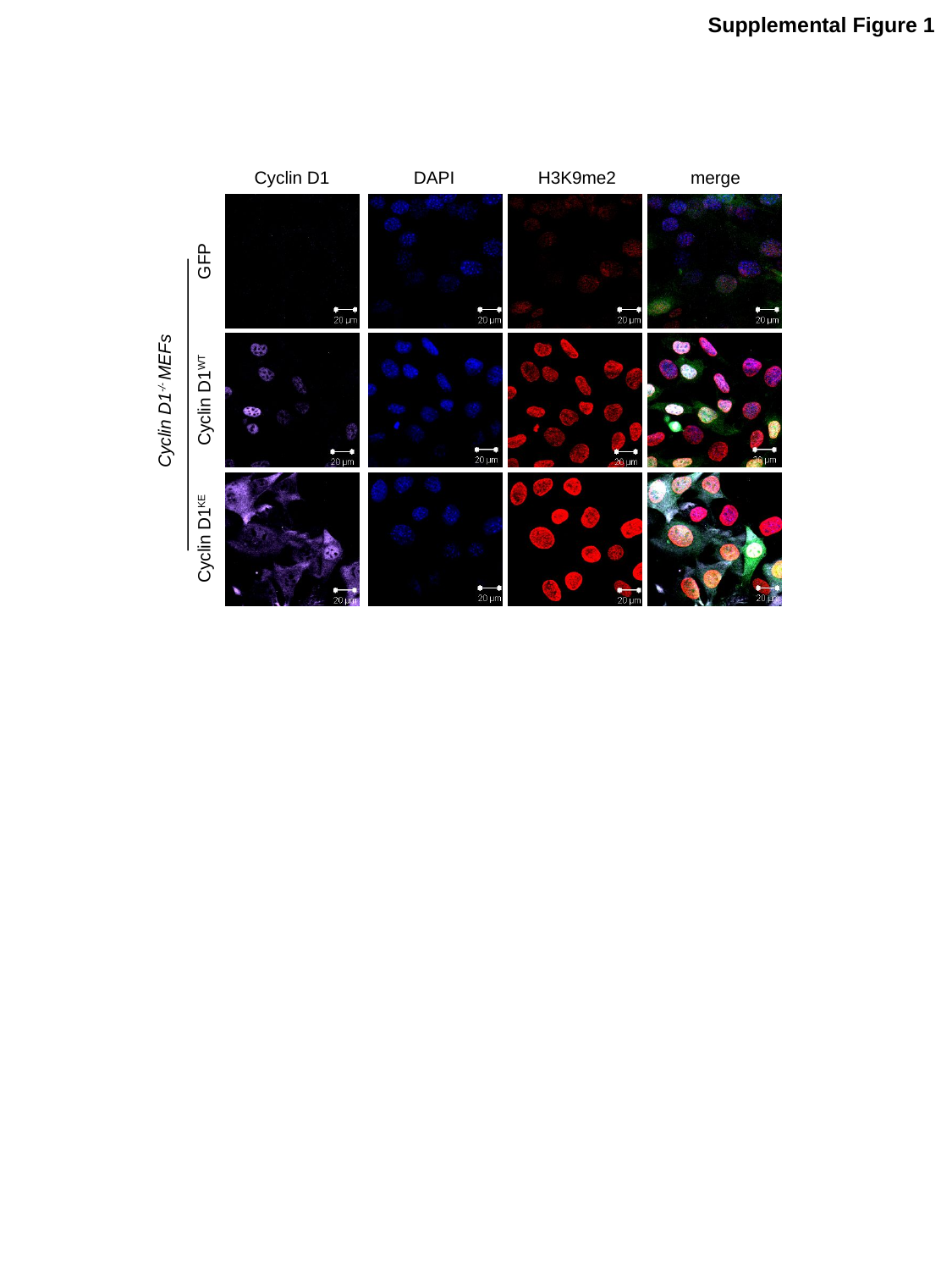

Supplemental Figure 1
Cyclin D1
DAPI
H3K9me2
merge
GFP
Cyclin D1WT
Cyclin D1-/- MEFs
Cyclin D1KE

## Slide 2
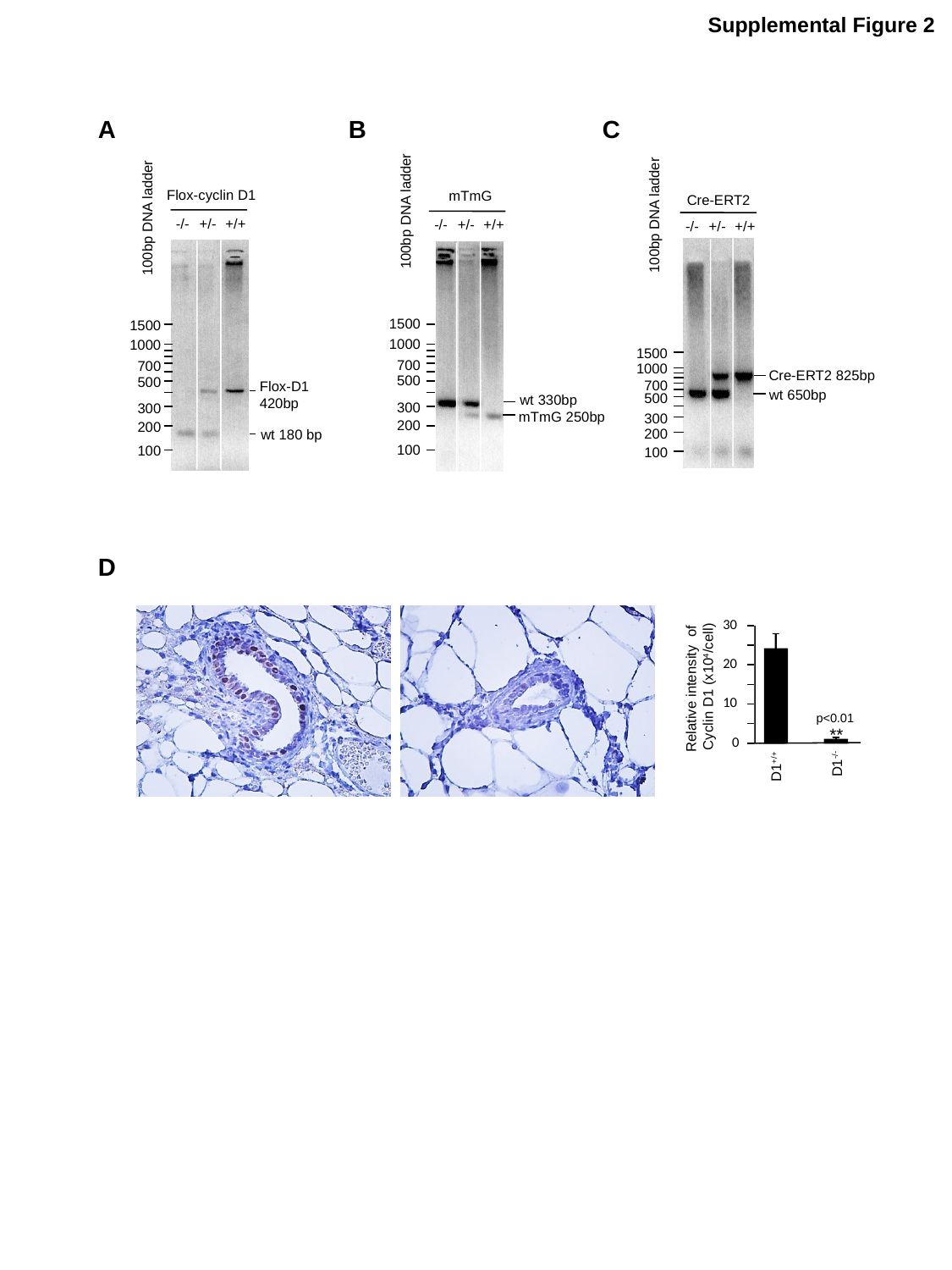

Supplemental Figure 2
A
B
C
Flox-cyclin D1
mTmG
Cre-ERT2
100bp DNA ladder
100bp DNA ladder
100bp DNA ladder
-/-
+/-
+/+
-/-
+/-
+/+
-/-
+/-
+/+
1500
1500
1000
1000
1500
1000
700
500
300
200
100
700
700
Cre-ERT2 825bp
500
500
Flox-D1
420bp
wt 650bp
wt 330bp
300
300
mTmG 250bp
200
200
wt 180 bp
100
100
D
30
20
Relative intensity of Cyclin D1 (x104/cell)
10
p<0.01
**
0
D1-/-
D1+/+

## Slide 3
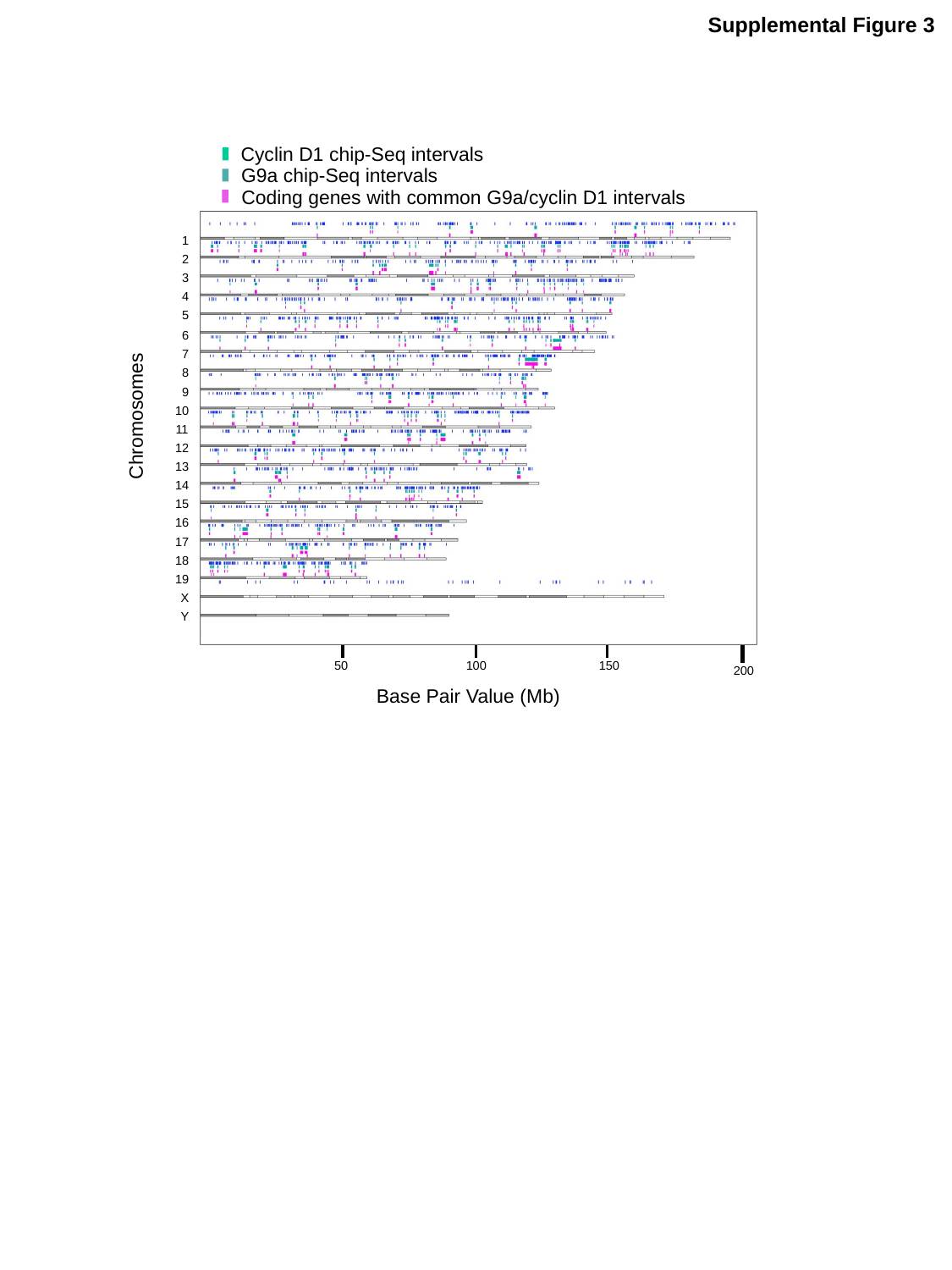

Supplemental Figure 3
Cyclin D1 chip-Seq intervals
G9a chip-Seq intervals
Coding genes with common G9a/cyclin D1 intervals
1
2
3
4
5
6
7
8
9
10
Chromosomes
11
12
13
14
15
16
17
18
19
X
Y
50
100
150
200
Base Pair Value (Mb)

## Slide 4
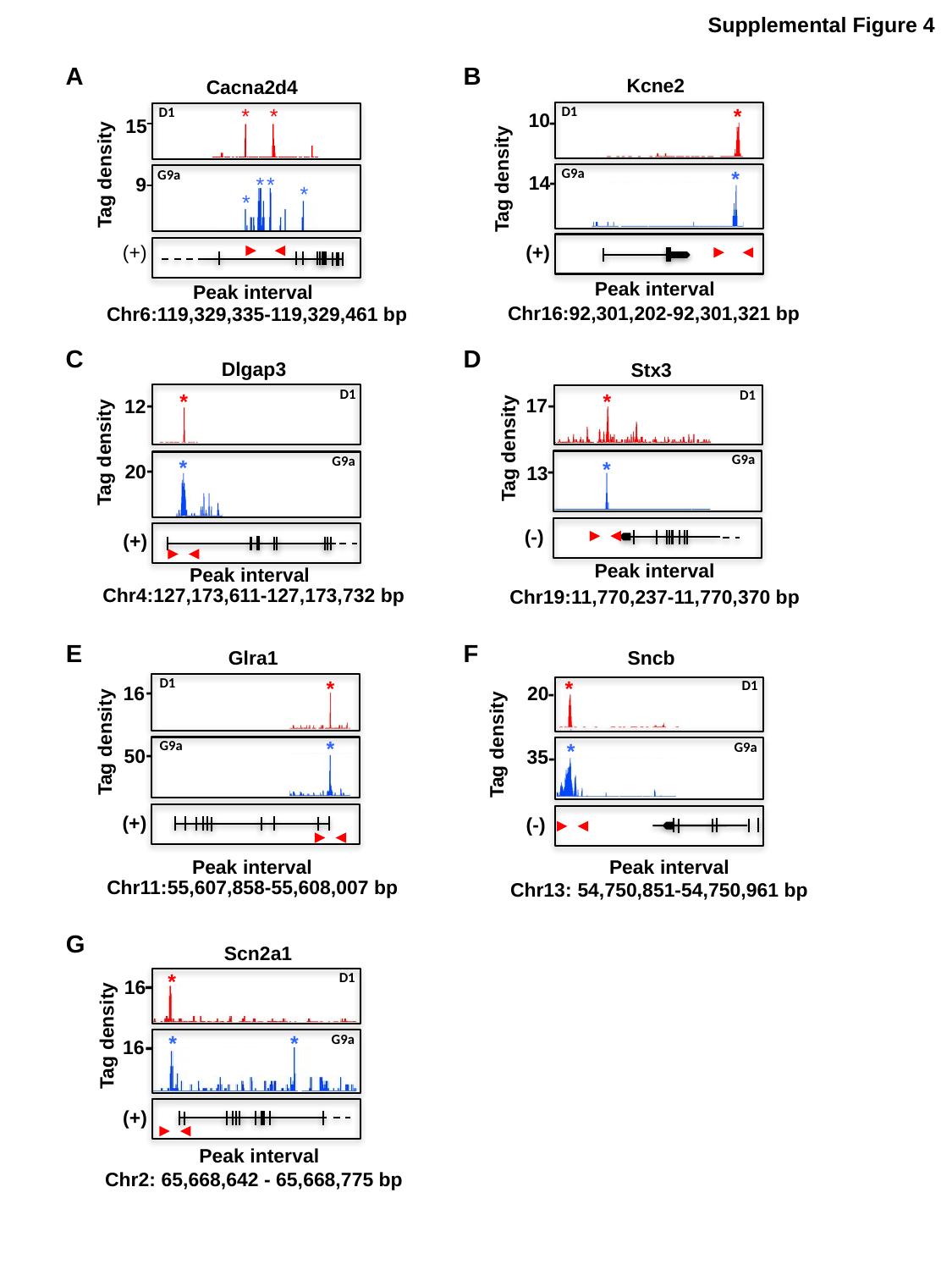

Supplemental Figure 4
B
A
Kcne2
Cacna2d4
*
*
*
10
D1
D1
-
-
15
Tag density
*
Tag density
-
G9a
14
-
*
*
G9a
9
*
*
(+)
(+)
Peak interval
Peak interval
Chr16:92,301,202-92,301,321 bp
Chr6:119,329,335-119,329,461 bp
C
D
Dlgap3
Stx3
*
*
D1
-
D1
-
17
12
Tag density
Tag density
*
*
G9a
-
G9a
-
20
13
(-)
(+)
Peak interval
Peak interval
Chr4:127,173,611-127,173,732 bp
Chr19:11,770,237-11,770,370 bp
E
F
Glra1
Sncb
*
*
-
D1
-
16
20
D1
Tag density
Tag density
*
*
-
G9a
50
G9a
35
-
(+)
(-)
Peak interval
Peak interval
Chr11:55,607,858-55,608,007 bp
Chr13: 54,750,851-54,750,961 bp
G
Scn2a1
*
-
D1
16
Tag density
*
*
-
16
G9a
(+)
Peak interval
Chr2: 65,668,642 - 65,668,775 bp

## Slide 5
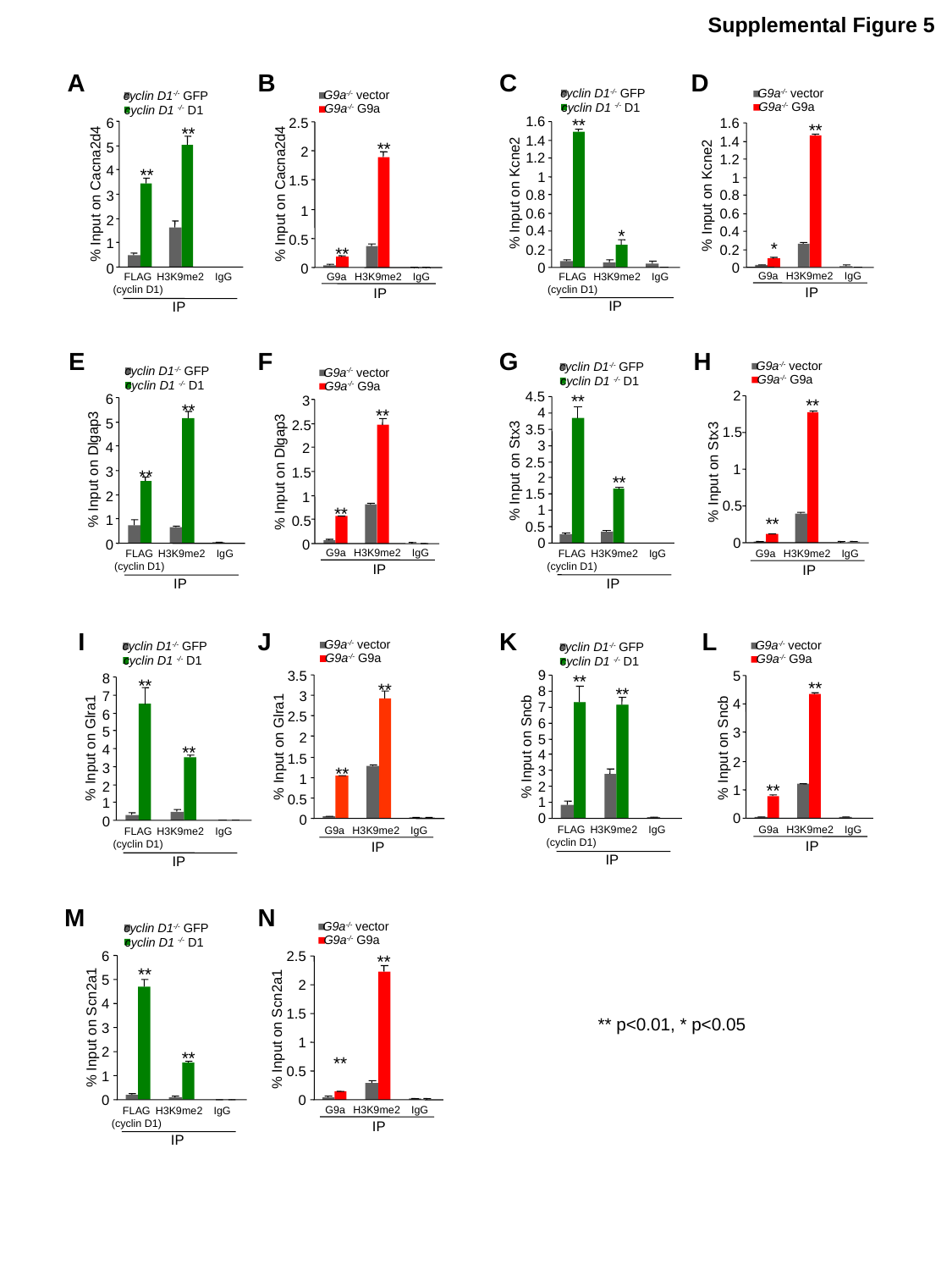

Supplemental Figure 5
A
B
C
D
cyclin D1-/- GFP
cyclin D1 -/- D1
G9a-/- vector
G9a-/- vector
G9a-/- G9a
cyclin D1-/- GFP
cyclin D1 -/- D1
G9a-/- G9a
**
**
1.6
6
1.6
2.5
**
**
1.4
1.4
5
2
1.2
1.2
**
4
1
1
1.5
0.8
0.8
3
% Input on Kcne2
% Input on Cacna2d4
% Input on Cacna2d4
% Input on Kcne2
1
0.6
0.6
2
*
0.4
0.4
0.5
*
1
**
0.2
0.2
0
0
0
0
G9a
H3K9me2
IgG
IP
FLAG (cyclin D1)
H3K9me2
IgG
IP
G9a
H3K9me2
IgG
IP
FLAG (cyclin D1)
H3K9me2
IgG
IP
E
F
G
H
G9a-/- vector
G9a-/- G9a
cyclin D1-/- GFP
cyclin D1 -/- D1
cyclin D1-/- GFP
cyclin D1 -/- D1
G9a-/- vector
G9a-/- G9a
**
4.5
4
3.5
3
2.5
**
2
1.5
1
0.5
2
**
1.5
1
% Input on Stx3
0.5
**
0
6
3
**
**
5
2.5
4
2
**
3
% Input on Dlgap3
% Input on Stx3
1.5
% Input on Dlgap3
2
1
**
1
0.5
0
0
0
G9a
H3K9me2
IgG
IP
FLAG (cyclin D1)
H3K9me2
IgG
IP
G9a
H3K9me2
IgG
IP
FLAG (cyclin D1)
H3K9me2
IgG
IP
I
J
K
L
G9a-/- vector
G9a-/- G9a
G9a-/- vector
G9a-/- G9a
cyclin D1-/- GFP
cyclin D1 -/- D1
cyclin D1-/- GFP
cyclin D1 -/- D1
**
9
**
8
7
6
5
% Input on Sncb
4
3
2
1
0
5
**
4
3
% Input on Sncb
2
**
1
0
3.5
**
3
2.5
2
% Input on Glra1
1.5
**
1
0.5
0
**
8
7
6
5
**
4
% Input on Glra1
3
2
1
0
G9a
H3K9me2
IgG
IP
FLAG (cyclin D1)
H3K9me2
IgG
IP
G9a
H3K9me2
IgG
IP
FLAG (cyclin D1)
H3K9me2
IgG
IP
M
N
G9a-/- vector
G9a-/- G9a
cyclin D1-/- GFP
cyclin D1 -/- D1
**
2.5
2
1.5
% Input on Scn2a1
1
**
0.5
0
6
**
5
4
3
% Input on Scn2a1
**
2
1
0
** p<0.01, * p<0.05
G9a
H3K9me2
IgG
IP
FLAG (cyclin D1)
H3K9me2
IgG
IP

## Slide 6
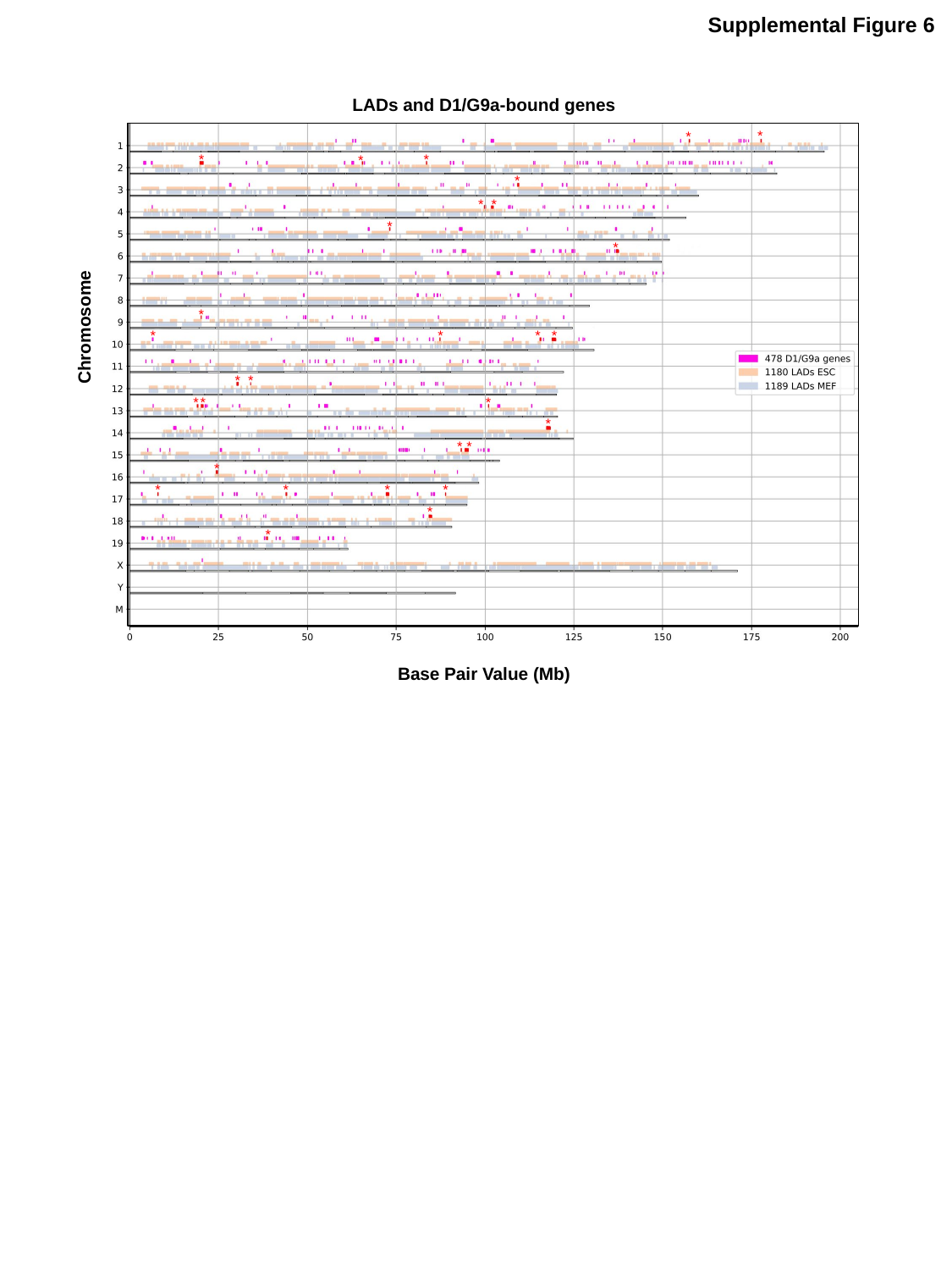

Supplemental Figure 6
LADs and D1/G9a-bound genes
*
*
*
*
*
*
*
*
*
*
*
Chromosome
*
*
*
*
*
*
*
*
*
*
*
*
*
*
*
*
*
*
*
Base Pair Value (Mb)

## Slide 7
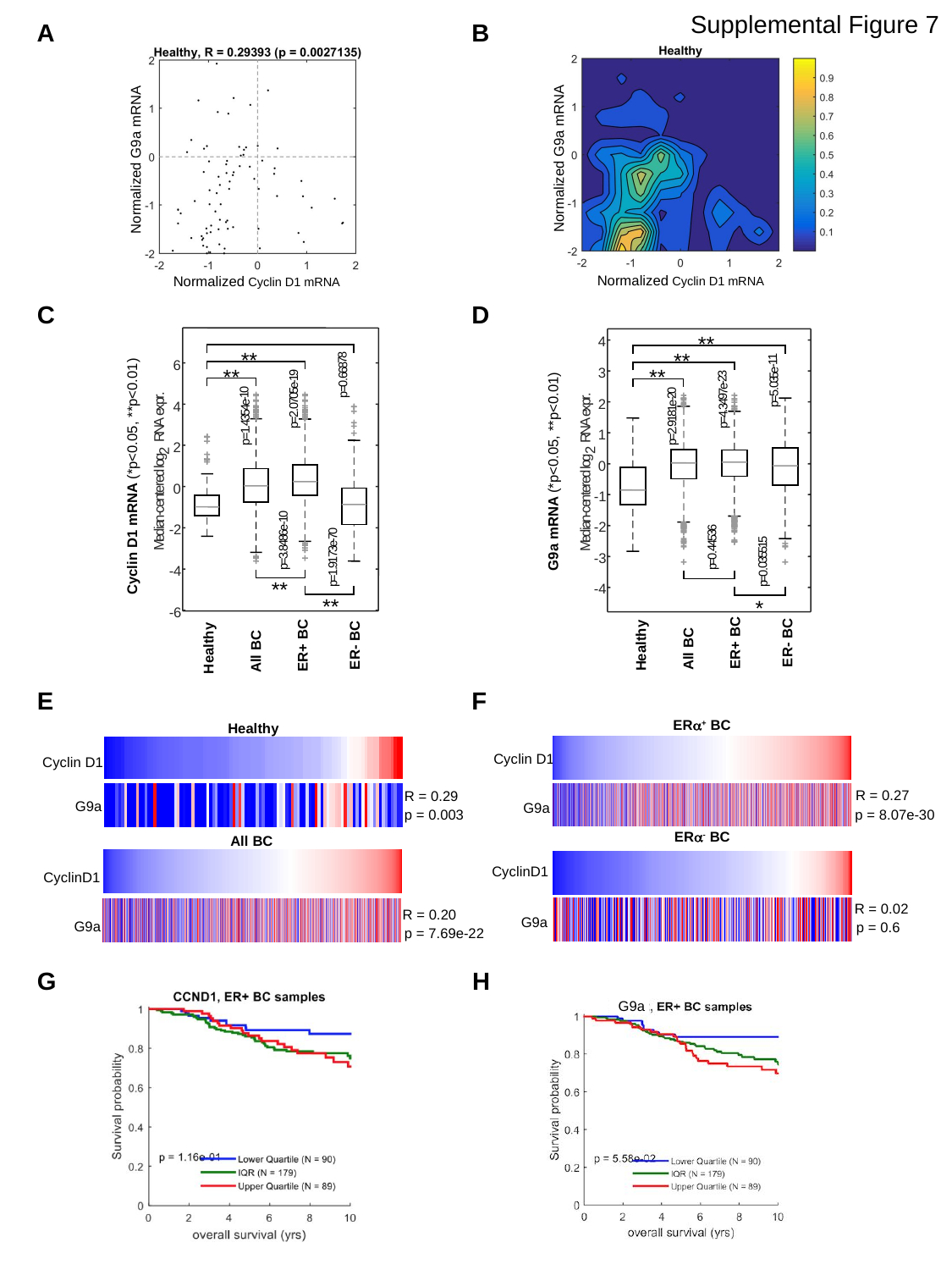

Supplemental Figure 7
B
A
Normalized G9a mRNA
Normalized Cyclin D1 mRNA
Normalized G9a mRNA
Normalized Cyclin D1 mRNA
C
D
**
4
**
1
1
**
-
3
e
3
5
2
3
-
0
e
.
0
7
5
.
2
9
r
=
-
2
p
4
e
p
x
3
1
.
e
8
4
1
=
A
9
p
.
N
1
2
=
R
p
2
g
0
o
l
G9a mRNA (*p<0.05, **p<0.01)
d
e
r
e
t
-1
n
e
c
-
n
a
-2
6
i
d
3
e
5
5
4
M
1
4
5
.
5
-3
0
3
=
0
p
.
0
=
p
-4
*
ER- BC
ER+ BC
Healthy
All BC
**
8
7
6
**
8
6
9
6
1
.
-
0
e
=
0
5
.
p
1
0
r
-
p
7
e
4
x
0
4
.
e
5
2
3
=
A
4
p
.
N
1
=
R
p
2
2
g
o
l
d
Cyclin D1 mRNA (*p<0.05, **p<0.01)
e
r
0
e
t
n
e
c
-
0
n
1
a
-
-2
i
e
0
d
6
7
e
8
-
4
M
e
8
3
.
7
3
1
=
9
p
-4
.
1
**
=
p
**
-6
ER- BC
ER+ BC
Healthy
All BC
E
F
ERa+ BC
Cyclin D1
 R = 0.27
G9a
 p = 8.07e-30
ERa- BC
CyclinD1
 R = 0.02
G9a
 p = 0.6
Healthy
Cyclin D1
 R = 0.29
G9a
 p = 0.003
All BC
CyclinD1
 R = 0.20
G9a
 p = 7.69e-22
G
H
 G9a

## Slide 8
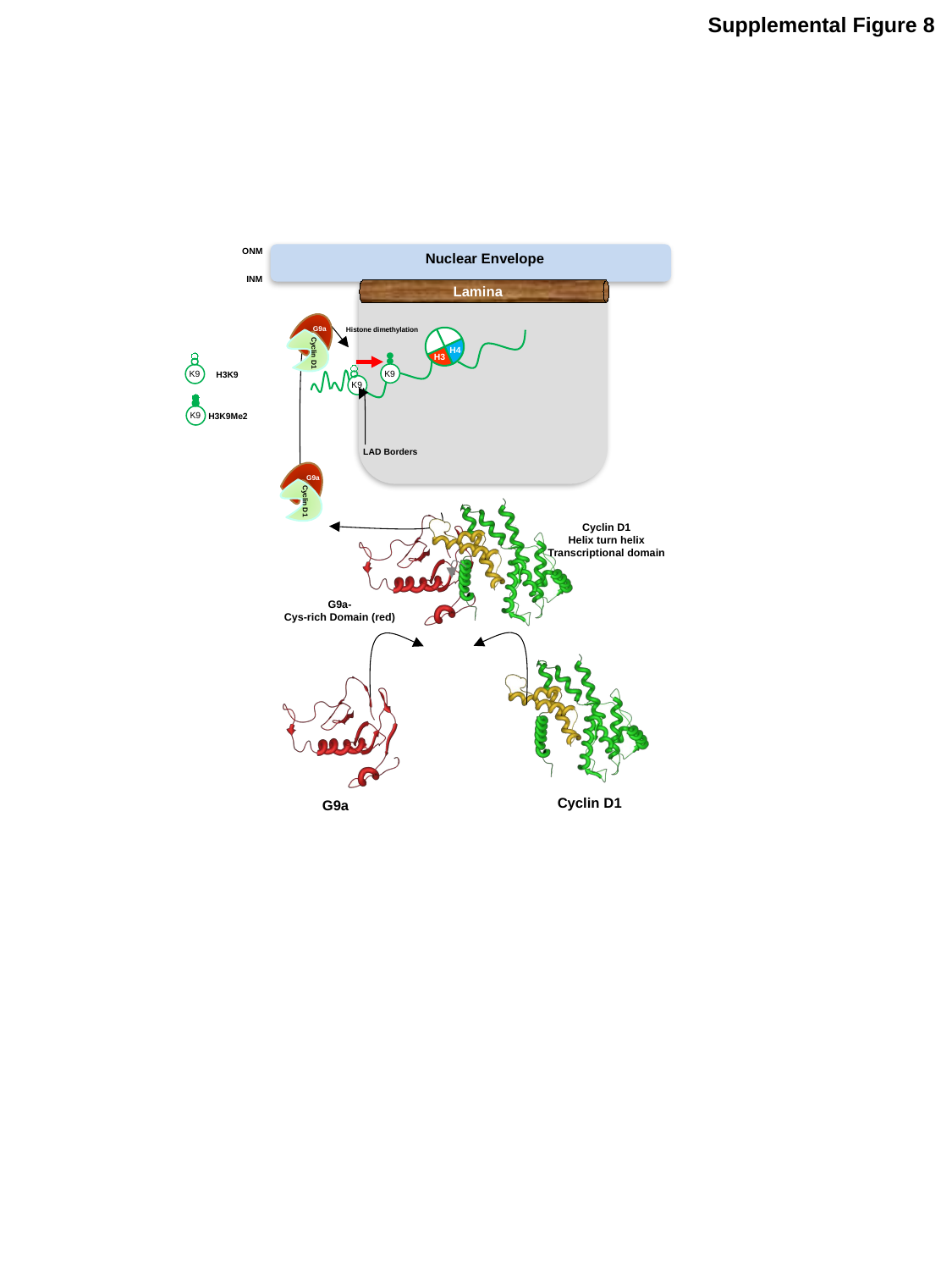

Supplemental Figure 8
ONM
Nuclear Envelope
INM
Lamina
G9a
Histone dimethylation
H4
Cyclin D1
H3
K9
K9
H3K9
K9
K9
H3K9Me2
LAD Borders
G9a
Cyclin D1
Cyclin D1
Helix turn helix
Transcriptional domain
G9a-
Cys-rich Domain (red)
Cyclin D1
G9a
